# Supplementary material for: Arrow heads at Obi-Rakhmat (Uzbekistan) 80 ka ago?
Source: PLoS One. 2025 Aug 11;20(8):e0328390. doi: 10.1371/journal.pone.0328390 (PMC12338843; doi:10.1371/journal.pone.0328390)
Supplement: S1 Note — (DOCX) [file pone.0328390.s001.docx]

S1 Note

**Supporting information for:**

Arrow heads at Obi-Rakhmat (Uzbekistan) 80 ka ago?

Hugues Plisson, Alëna V. Kharevich, Vladimir M. Kharevich, Pavel V. Chistiakov, Lydia V. Zotkina, Malvina Baumann, Eric Pubert, Ksenya A. Kolobova, Farhod A. Maksudov, Andrei I. Krivoshapkin

**Additional methodological observation**

Based on their impact damage, the points identified as weapon heads can be characterised on two levels.

The first focuses on the information provided by the impact traces, according to the following criteria:

- the morphology of the damage, which reflects the orientation of the stress in relation with the delivery system (Coppe et al., 2023; Coppe and Rots, 2017).

- the extent of the damage, which depends on the energy dissipated on impact, resulting from the momentum of the projectile [« Momentum determines the amount of force which an arrow has available to it for penetration » (Ashby, 2005, p. 12)], the hardness of the impacted material, the impact angle and the toughness of the attachment of the point to the shaft, according to the material, shape and dimensions of the point or insert.

- the location of the damage, depending on the position on the shaft and the robustness of the hafting (eg. Plisson, 2005).

Since the shape, size and material of the point, as well as its attachment to the shaft affect these criteria, it is not a valid approach to transfer values from one study to another on the basis of the literature alone. For example, the lateral edges of a narrow point will be less crushed than those of a point whose width exceeds the intercostal space, while the fracture termination will be longer on a piece with a triangular cross-section than on one with an elliptical cross-section.

Experimental calibration is therefore necessary in order to adapt the criteria to the specificities of the archaeological collection being studied.

However, the experiment must not be too far from the real technical conditions. Ballistic gelatine targets do not reproduce the anatomical structural complexity nor the physical characteristics of the different layers of organic tissue, fibrous and non-fibrous, of distinct cohesion, elasticity and viscosity, to be passed through before reaching a vital organ (Ashby, 2005). The uniformity of ballistic gelatine is suitable for comparing the penetration of firearm projectiles, i.e. conical-cylindrical projectiles shot with high kinetic energy (e.g. 630 J for a 9 mm bullet), for which it has been designed, but not for assessing the capacity of slow projectiles with low kinetic energy (e.g. 20 J for a 25 g arrow, or 63 J for a 200 g dart) whose penetration in real conditions depends essentially on their sharpness. Such “homogenous targets are not scalable to soft tissues; they tend to produce greater friction on larger surfaces and do not adequately capture the effects of sharper tips and edges”(Pettigrew et al., 2023). Furthermore, the uniformity of ballistic gelatine does not allow to observe the ability of the arrows points (more or less depending on their shape) to deviate from their penetration trajectory by sliding on the bones, due to the low inertia of their shafts, unlike javelins, which in realistic conditions results in a lower rate and amplitude of fracturing (*Le temps des chasseurs solutréens*, 1998).

The second level of analysis concerns interdependent functional parameters related to weapon structure, ballistic properties and lethal potential. These parameters have in common that they lead to a strict morphological and dimensional design.

On the one hand, for thrusted spears and short-range killing, the design emphasizes overall reliability, with particular attention paid to the strength of the junction between the armature and the shaft (Bleed, 1986) due to the high energy involved and the hunter's exposure; on the other hand, for long-range projectiles, ballistic accuracy and head acuteness are priorities (Hughes, 1998).

In the high-risk, high-return activity that hunting typically is, any reduction in uncertainty is essential. This means a design that ensures the durability of thrusting weapons or the reproducibility of arrow or dart shots, qualities that are linked to a strict compliance with physical principles that leave little room for improvisation (Ashby, 1987; Hughes, 1998). The nature of the game, its ethology, reactivity, aggressiveness and resilience are also among the parameters to be taken into account in this complex system.

Put simply, the design of a point will be very different depending on whether you're hunting rabbits with a bow or aurochs with a spear.

Several pieces of the archaeological puzzle are missing, but just as in palaeontology, where the shape of a tooth reveals the type of diet and therefore suggests the mode of locomotion, the characteristics of an armature provide information on the type of weapon for which it was the wounding element.

References

Ashby, E., 2005. Ashby Bowhunting Reports - Momentum, Kinetic Energy, and Arrow Penetration (And What They Mean for the Bowhunter), Ashby Bowhunting Reports.

Ashby, E., 1987. Ashby Bowhunting Reports [WWW Document]. Ashby Bowhunting Foundation. URL https://www.ashbybowhunting.org/ashby-reports (accessed 7.17.24).

Bleed, P., 1986. The Optimal Design of Hunting Weapons: Maintainability or Reliability. American Antiquity 51, 737–747. https://doi.org/10.2307/280862

Coppe, J., Rots, V., 2017. Focus on the target. The importance of a transparent fracture terminology for understanding projectile points and projecting modes. Journal of Archaeological Science: Reports 12, 109–123. https://doi.org/10.1016/j.jasrep.2017.01.010

Coppe, J., Taipale, N., Rots, V., 2023. Terminal ballistic analysis of impact fractures reveals the use of spearthrower 31 ky ago at Maisières-Canal, Belgium. Sci Rep 13, 18305. https://doi.org/10.1038/s41598-023-45554-w

Hughes, S.S., 1998. Getting to the point: Evolutionary change in prehistoric weaponry. J Archaeol Method Theory 5, 345–408. https://doi.org/10.1007/BF02428421

Le temps des chasseurs solutréens, 1998. . ADDC Archéolud ; Delta image ; France 3 Aquitaine ; Vision Age.

Pettigrew, D.B., Garnett, J., Ryals-Luneberg, C., Vance, E.A., 2023. Terminal Ballistics of Stone-Tipped Atlatl Darts and Arrows: Results From Exploratory Naturalistic Experiments. Open Archaeology 9. https://doi.org/10.1515/opar-2022-0299

Plisson, H., 2005. Examen tracéologique des pointes aziliennes du Bois-Ragot, in: Chollet, A., Dujardin, V. (Eds.), La Grotte Du Bois-Ragot à Gouex (Vienne).Magdalénien et Azilien. Essais Sur Les Hommes et Leur Environnement, Mémoire XXXVIII de La Société Préhistorique Française. Société Préhistorique Française, Paris, pp. 183–189.
