# Supplementary material for: Arrow heads at Obi-Rakhmat (Uzbekistan) 80 ka ago?
Source: PLoS One. 2025 Aug 11;20(8):e0328390. doi: 10.1371/journal.pone.0328390 (PMC12338843; doi:10.1371/journal.pone.0328390)
Supplement: S1 Table — (PDF) [file pone.0328390.s002.pdf]

Obi-Rakhmat, layers 20-21

Technological inventory

	20			21,1			21,2			21,3		
	N	%	%*	N	%	%*	N	%	%*	N	%	%*
**Cores**	14	0,42%	**1,64%**	72	1,30%	**2,49%**	9	0,46%	**1,41%**	2	0,34%	**0,97%**
**Core-like**	5	0,15%	**0,59%**	43	0,77%	**1,48%**	2	0,10%	**0,31%**	6	1,01%	**2,91%**
**Technical flakes**	21	0,6%	**2,5%**	44	0,8%	**1,5%**	17	0,9%	**2,7%**	8	1,3%	**3,9%**
**Blades**	280	8,5%	**32,8%**	1265	22,8%	**43,7%**	216	11,1%	**33,9%**	64	10,7%	**31,1%**
**Bladelets**	257	7,8%	**30,1%**	613	11,0%	**21,2%**	152	7,8%	**23,9%**	44	7,4%	**21,4%**
**Microblades**	56	1,7%	**6,6%**	109	2,0%	**3,8%**	9	0,5%	**1,4%**		0,0%	**0,0%**
**Points**	12	0,4%	**1,4%**	120	2,2%	**4,1%**	43	2,2%	**6,8%**	19	3,2%	**9,2%**
**Flakes > 3 cm**	208	6,3%	**24,4%**	630	11,4%	**21,8%**	189	9,7%	**29,7%**	63	10,6%	**30,6%**
Including Tools:	*32*	1,0%	**3,8%**	*209*	3,8%	**7,2%**	*34*	1,7%	**5,3%**	*9*	1,5%	**4,4%**
**Flakes 1–3 cm**	589	17,9%	**-**	1533	27,6%	**-**	1116	57,1%	**-**	304	50,9%	**-**
**Chunks/shatter**	48	1,5%	**-**	57	1,0%	**-**	34	1,7%	**-**	19	3,2%	**-**
**Chips**	1806	54,8%	**-**	1064	19,2%	**-**	166	8,5%	**-**	68	11,4%	**-**
	**3296**	**100,0%**		**5550**	**100,0%**		**1953**	**100,0%**		**597**	**100,0%**	

Table. Overview of the lithic assemblage

CORES	20		21,1		21,2		21,3	
	**N**	**%**	**N**	**%**	**N**	**%**	**N**	%
**Blade cores**	2	14,3%	24	33,8%	2	22,2%	1	50,0%
**Levallois (points cores)**			4	5,6%	3	33,3%		
**Levallois (flakes cores)**	1	7,1%	5	7,0%				
**Bladelet cores**	7	50,0%	25	35,2%	3	33,3%		
**Flake cores (other systems)**	4	28,6%	13	18,3%	1	11,1%	1	50,0%
**Total**	14		71	100,0%	9	100,0%	2	100,0%

Table. Type of determinable cores (core-like pieces are excluded)

CORES	20		21,1		21,2		21,3	
	**N**	**%**	**N**	**%**	**N**	**%**	**N**	**%**
**Flat faced, Unidirectional**	-	-	6	25%	-	-	1	100%
**Flat faced, Bidirectional**	-	-	3	13%	-	-	-	-
**Sub-prismatic Unidirectional**	-	-	-	-	2	100%	-	-
**Sub-prismatic Bidirectional**	-	-	1	4%	-	-	-	-
**Sub-prismatic Asymmetrical (*Semi-tourné*)**	-	-	2	8%	-	-	-	-
**Narrow-faced**	2	100%	12	50%	-	-	-	-
**Total**	**2**	**100%**	**24**	**100%**	**2**	**100%**	**1**	**100%**

Table. Type of Blade cores

CORES	20		21,1		21,2		21,3	
	**N**	**%**	**N**	**%**	**N**	**%**	**N**	%
**Carenated**			4	16,0%				
**Narrow-faced**	1	14,3%	4	16,0%	2	66,7%	0	
**Burin-cores**	3	42,9%	5	20,0%				
**Flat faced**	1	14,3%						
**Sub-prismatic**			7	28,0%				
**Other core on flakes**	2	28,6%	5	20,0%	1	33,3%		
**Total**	7	100,0%	25	100,0%	3	100,0%	0	

Table. Bladelet cores

S2 Table
Supporting information for:
Arrow heads at Obi-Rakhmat (Uzbekistan) 80 ka ago?
