# Supplementary material for: Arrow heads at Obi-Rakhmat (Uzbekistan) 80 ka ago?
Source: PLoS One. 2025 Aug 11;20(8):e0328390. doi: 10.1371/journal.pone.0328390 (PMC12338843; doi:10.1371/journal.pone.0328390)
Supplement: S2 Table — (PDF) [file pone.0328390.s003.pdf]

S2 Table

Supporting information for:

Arrow heads at Obi-Rakhmat (Uzbekistan) 80 ka ago?

№	Distance (m)	Localisation of the impact	Impacted anatomical part	Impact damage of arrow points	Macro fracture	
1	8	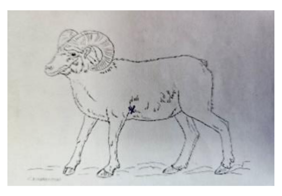	Arrow hit the sternum	Broke off from the arrow shaft	**Distal part fractured, proximal part shows step-fracture**	
2	5	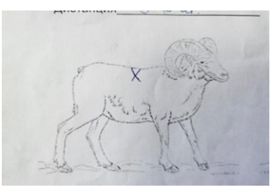	The arrow passed through the spine	The arrowhead broke	**micro step-termination / snap with tiny step**	
3	5	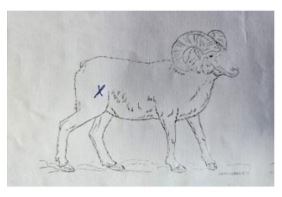	The arrow pierced through the muscles of the left leg and struck the bone of the right leg	no visible damage	**Hinge-termination (ventral part)**	
4	8	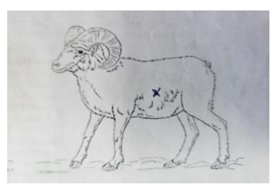	The broadhead passed completely through the abdomen	No visible cracks, except for a minor flake scar (tongue-shaped fracture)	**tiny hinge termination**	
5	8	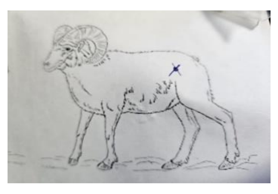	Complete pass-through (exit wound visible)	no visible damage	no impact	
6	8	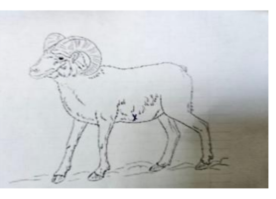	The arrowhead passed completely through the abdomen	no visible damage	no impact	
9	6	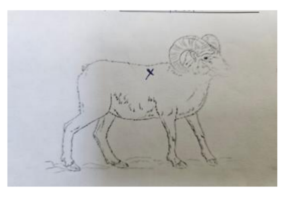	1st shot missed (hit the backstop). 2nd shot - complete pass-through entry at the nape, with bone contact	Tongue-shaped flake scar	**Complex fracture (distal part, ventral)**	
10	8	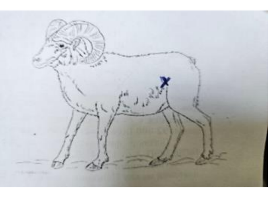	The arrow hit the abdomen near the leg and passed right through	no visible damage	no impact	
11	8	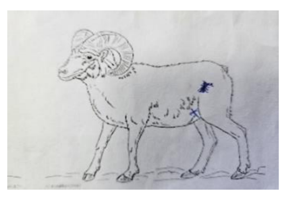	Soft-tissue penetration - no bone contact	no visible damage	no impact	
12	8	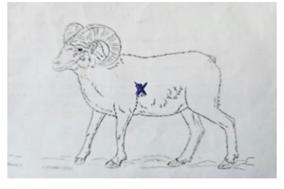	The arrow hit the shoulder blade or nearby, lodging between the ribs	no visible damage	no impact	
13	7	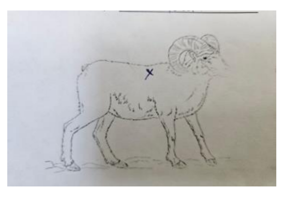	The arrow found its mark in the spine, the broadhead wedged tight between vertebrae	Micro-fracture at the tip	**tiny step-termination (dorsal part)**	
14	7	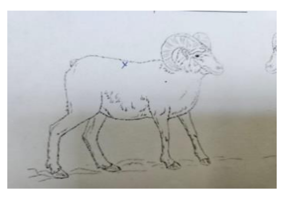	The arrow passed cleanly through the intervertebral space	no visible damage	no impact	
17	5	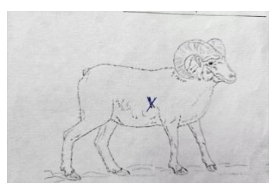	The projectile penetrated the peritoneum and became lodged in osseous tissue	The tip broke off	**Step-terminating fracture (ventral) + MLIT**	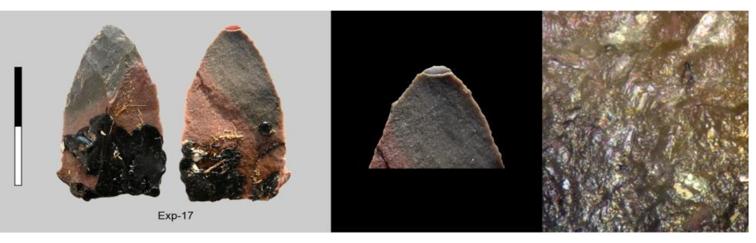
18	6	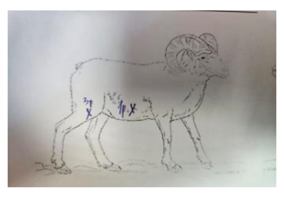	First shot: abdominal penetration (through-and-through). Second shot: missed, lodged in shield – tip intact. Third shot: leg hit – shaft broke, tip embedded in flesh with bone contact	First hit: no damage detected (clean pass-through) Second hit: minor tip fracture observed	**Snap termination**	
16	10		Stuck into the earthen-stone floor	The arrowhead broke	**Step / Complex termination**	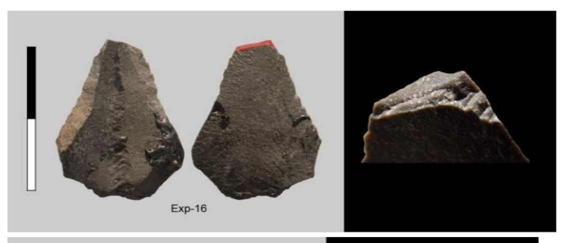
19	8	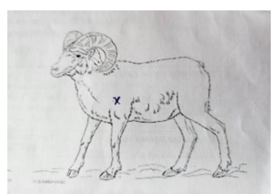	The arrow penetrated the left lateral torso and became trapped medially within the right scapula	The tip of the arrowhead broke off	**Step-terminating bending fracture (ventral)**	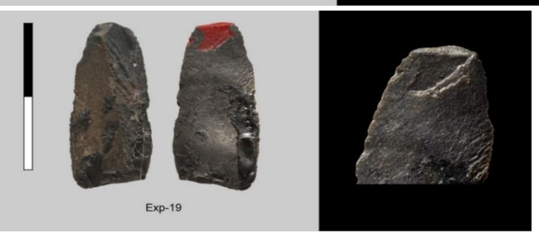
20	5	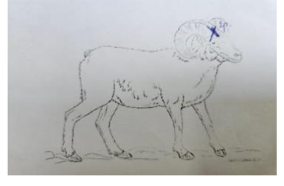	First shot missed, hit the backstop. Second one lodged in the scalp	no visible damage	no impact	
21	8	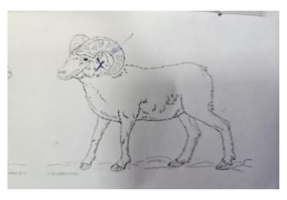	The initial shaft barely ruffled the animal's head fur. The follow-up hit was lethal - penetrating the cervical spine's natural gap	The point broke off	**Step-terminating bending fracture (dorsal) + MLIT?**	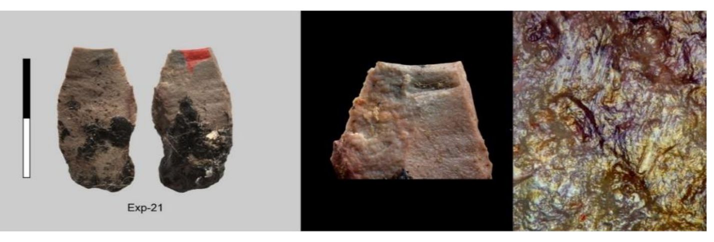
22	8	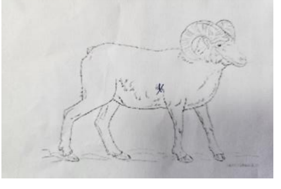	The arrow hit the ram's fleece near the hind leg, passing clean through the thick wool and muscle	no visible damage	no impact	
23	5	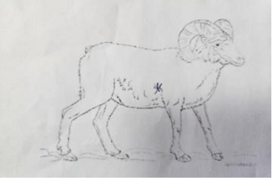	The arrow pierced right through the ram's belly, emerging from the opposite side.	no visible damage	**Proximal part fractured, micro scar on the edge**	
25	6	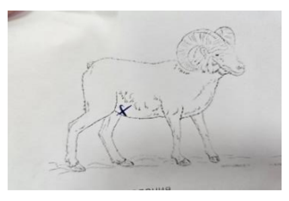	The arrow passed through soft tissue and grazed the femur of the left hind leg	The arrowhead broke	**Step-terminating fracture (dorsal)**	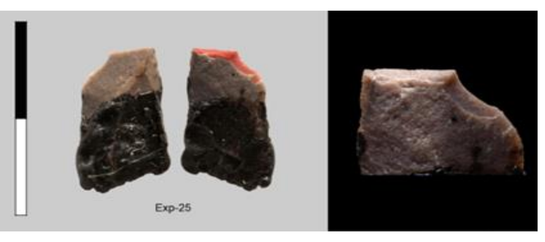

Experimental arrow heads in local silicified limestone: shot results.
