# Supplementary material for: Arrow heads at Obi-Rakhmat (Uzbekistan) 80 ka ago?
Source: PLoS One. 2025 Aug 11;20(8):e0328390. doi: 10.1371/journal.pone.0328390 (PMC12338843; doi:10.1371/journal.pone.0328390)
Supplement: S3 Table — (PDF) [file pone.0328390.s004.pdf]

S3 Table

Supporting information for:

Arrow heads at Obi-Rakhmat (Uzbekistan) 80 ka ago?

	Inventory	Typo/technology	Length (mm)	Width (mm)	Thickness (mm)	Weight (g)	Fragment	TCSA	TCSP
**Micro-points**									
0	OP Сл 21_X7	Levallois micropoint	21,8	17,3	3,0	1,1	complete	26,0	35,6
2	OP 21.1 280	retouched micropoint	29,8	20,7	5,6	4,5	2/3 prox.	58,0	44,2
4	OP-01 20	raw micropoint	13,0	15,0	3,9	1,0	1/2 prox.	29,3	31,9
5	OP 01-08 СЛ 21.1	Levallois micropoint	14,6	15,2	3,9	0,8	1/2 prox.	29,6	32,3
6	OP 2001-2008 СЛ 21.1	raw micropoint	13,4	16,4	3,4	0,7	1/2 prox.	27,9	34,2
7	OP СЛ 21.1	raw micropoint	21,7	18,3	4,9	2,1	2/3 prox.	44,8	39,1
8	OP 11 П 7 21.1	raw micropoint	17,7	17,6	3,8	1,1	1/2 prox.	33,4	36,8
9	OP 01 СЛ20.3	raw micropoint	19,2	23,7	5,3	2,5	1/2 prox.	62,8	49,7
11	OP 2001-2008 СЛ 21.1	Levallois micropoint	23,3	22,4	4,1	2,4	2/3 prox.	45,9	46,3
16	OP 2001-2008 СЛ 21.1	raw micropoint	17,5	15,7	3,9	1,0	2/3 prox.	30,6	33,2
Mean				18,2	4,2	1,7		38,8	38,3
Std Dev				2,9	0,8	1,1		12,6	6,0
Min.				15,0	3,0	0,7		26,0	31,9
Max.				23,7	5,6	4,5		62,8	49,7
**Large points**									
20	OP 10 21.1 0-6	retouched point	45,1	30,3	12,2	15,4	1/2 prox.	184,8	69,2
22	OP 11 21.1	retouched point	30,4	33,0	6,4	6,9	40% distal	105,6	
24	OP СЛ 21.1 196	retouched elongated point	72,6	34,4	11,2	33,6	90% prox.	192,6	75,5
26	OP 10 06 21.1 622	retouched point	64,2	41,0	8,6	25,0	almost complet	176,3	85,5
27	OP 11 П 7 21.1	retouched point	17,5	16,9	4,7	1,2	distal	39,7	
28	OP 11 21.2 06 КВ 145	retouched point	58,6	38,1	10,5	24,4	almost complet	200,0	81,6
30	OP 11 21.2 П6 17	retouched point	25,1	19,3	4,7	2,5	distal	45,4	
104	OP 01 19 4	retouched point	21.9	25,9	6,1	2,9	1/3 distal	79,0	

TCSA and TCSP calculated according to the formulas used by Sisk et Shea 2009 and Pettigrew et al. 2023 after Hughes 1998

**Bladelets**							
15	OP 11 21.1 07	bladelet	23,1	11,2	3,6	1,0	half prox.
19	OP 2001-2008 СЛ 21.1 П8	elongated point with tiny retouch	27,1	15,0	4,6	1,7	distal
31	ОП-08 Сл 21.1 КВ М7	backed bladelet	34,1	10,2	3,1		

3D models provided in Supporting information are millimeter-scaled

Weapon heads and inserts: morphometric data
