# Supplementary material for: Arrow heads at Obi-Rakhmat (Uzbekistan) 80 ka ago?
Source: PLoS One. 2025 Aug 11;20(8):e0328390. doi: 10.1371/journal.pone.0328390 (PMC12338843; doi:10.1371/journal.pone.0328390)
Supplement: S4 Table — (PDF) [file pone.0328390.s005.pdf]

S4 Table
Supporting information for:
Arrow heads at Obi-Rakhmat (Uzbekistan) 80 ka ago?

truncated-faceted	Cores	Bladelet cores	micropoints	Points
58	67	79	80	86
65	63	84	81	75
70	78	86	76	65
59	80	92	82	88
63	93	86	76	71
58	86	86	71	72
63	90	85	85	
70	87	77	71	
72	73	86	83	
81	96	84	79	
79	82	66	88	
54	82	78	61	
66	86	73		
60	93	78		
77	76	63		
78	65	83		
69	87	74		
76	83	82		
67	89	76		
67	95	82		
60	76	57		
64	85	80		
66		90		
58		69		
71		78		
54		79		
70		72		
73		57		
64		79		
70		85		
76		85		
82		79		
82				
78				
82				
74				
76				
71				
81				
66				
65				
76				
81				
82				

66
74
78
69
73
68
64
66
66
90
62
73
71
61
64
68
69
65
69
61
66
63
55
64
68
69
72
55
71
74
64
79
67
70
67
76
74
66
55
73
69
81
74
76
84
57
78
58
62
62

76
72
50
72
81
70
57
72
81
69
72
72
74
81
64
79
59
72
74
61
68
62
69
69
66
69
74
78

Raw data for Fig 15
