# Supplementary figures and images for: Arrow heads at Obi-Rakhmat (Uzbekistan) 80 ka ago?
Source: PLoS One. 2025 Aug 11;20(8):e0328390. doi: 10.1371/journal.pone.0328390 (PMC12338843; doi:10.1371/journal.pone.0328390)

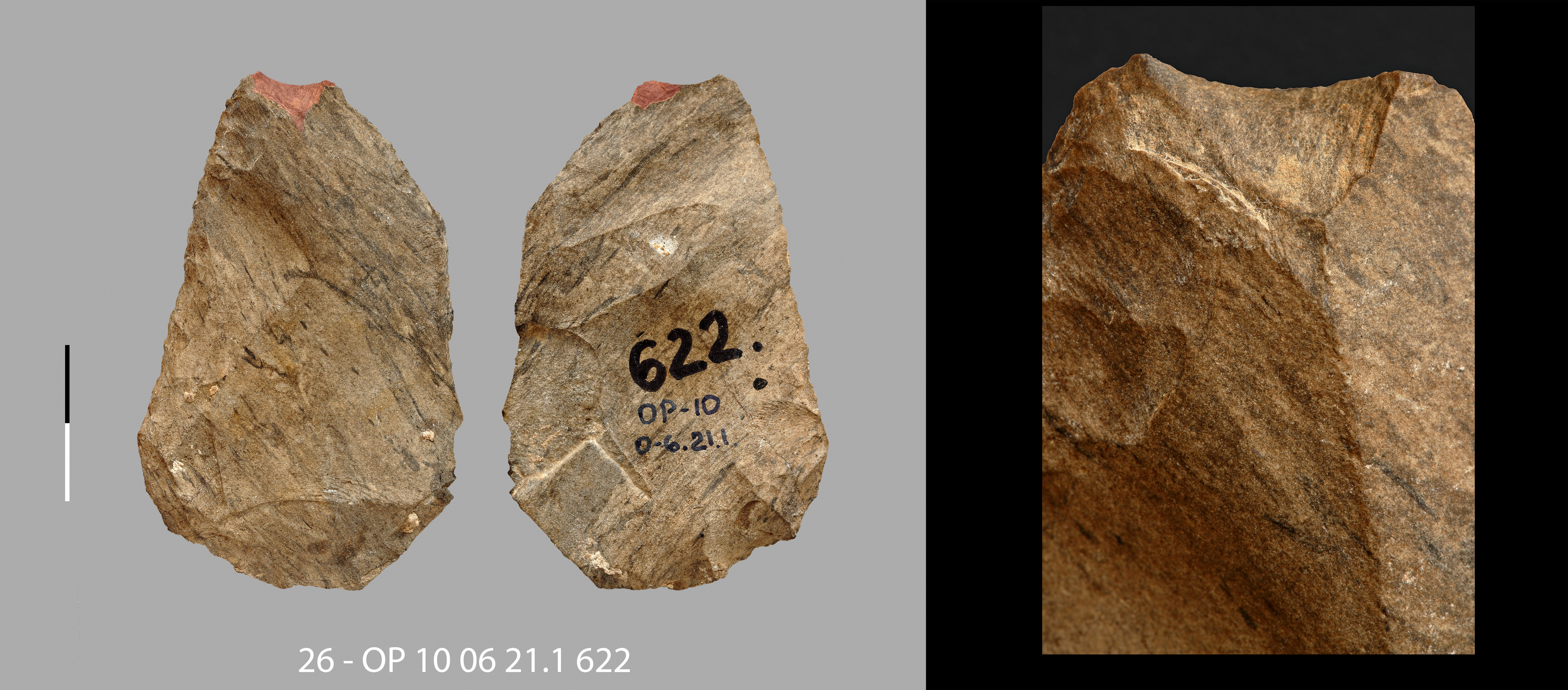

Supplement: S1 Fig — (JPG) [file pone.0328390.s006.jpg]

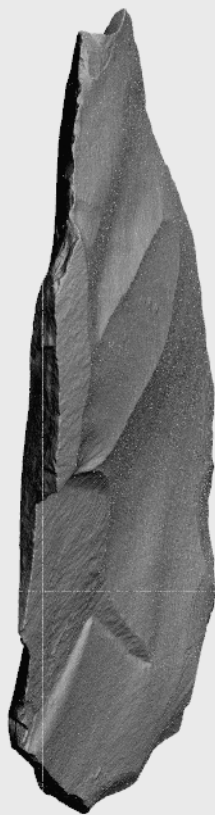

Obi-Rakhmat: 26 - OP 10 06 21.1 622

Supplement: S1 File — (PDF) [file pone.0328390.s007.pdf]

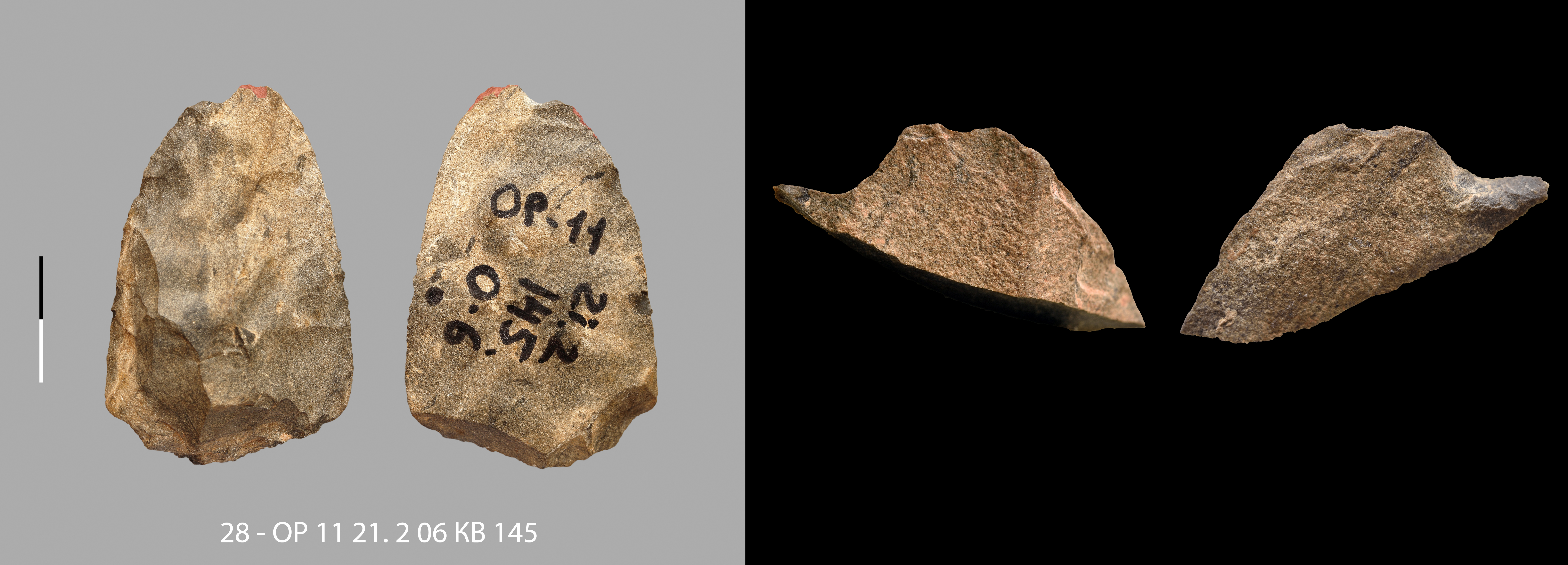

Supplement: S2 Fig — (JPG) [file pone.0328390.s008.jpg]

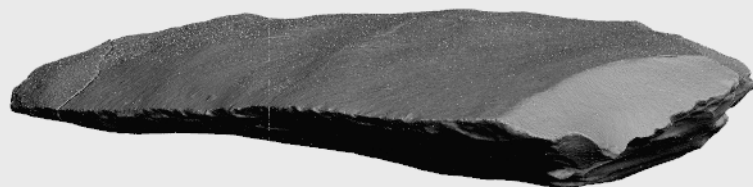

Supplement: S2 File — (PDF) [file pone.0328390.s009.pdf]

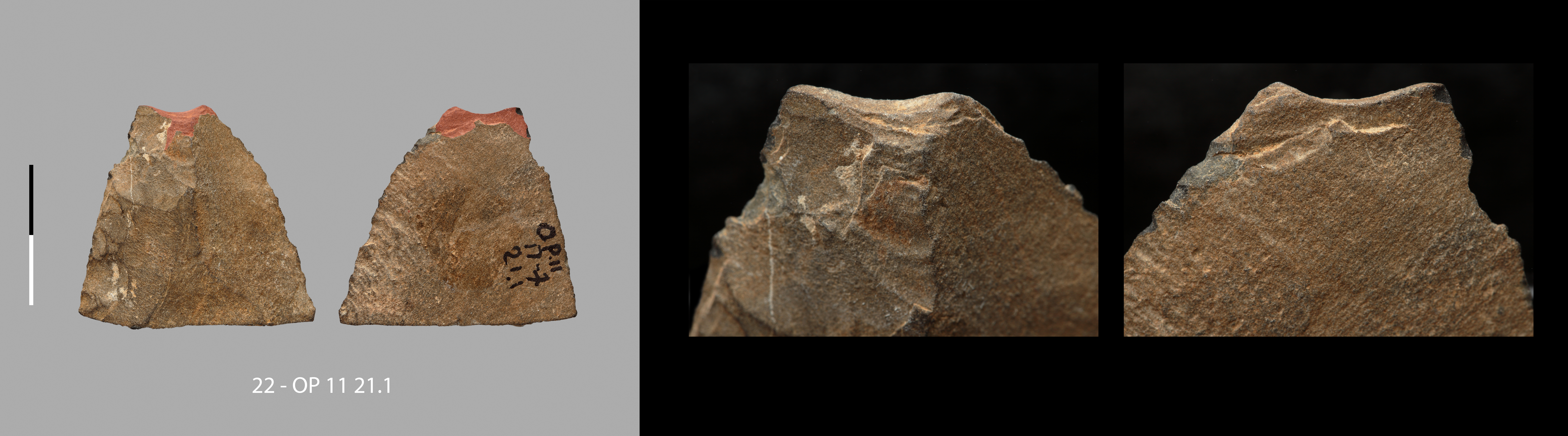

Supplement: S3 Fig — (JPG) [file pone.0328390.s010.jpg]

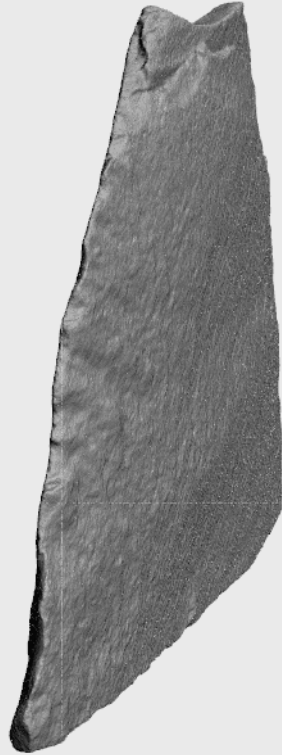

Supplement: S3 File — (PDF) [file pone.0328390.s011.pdf]

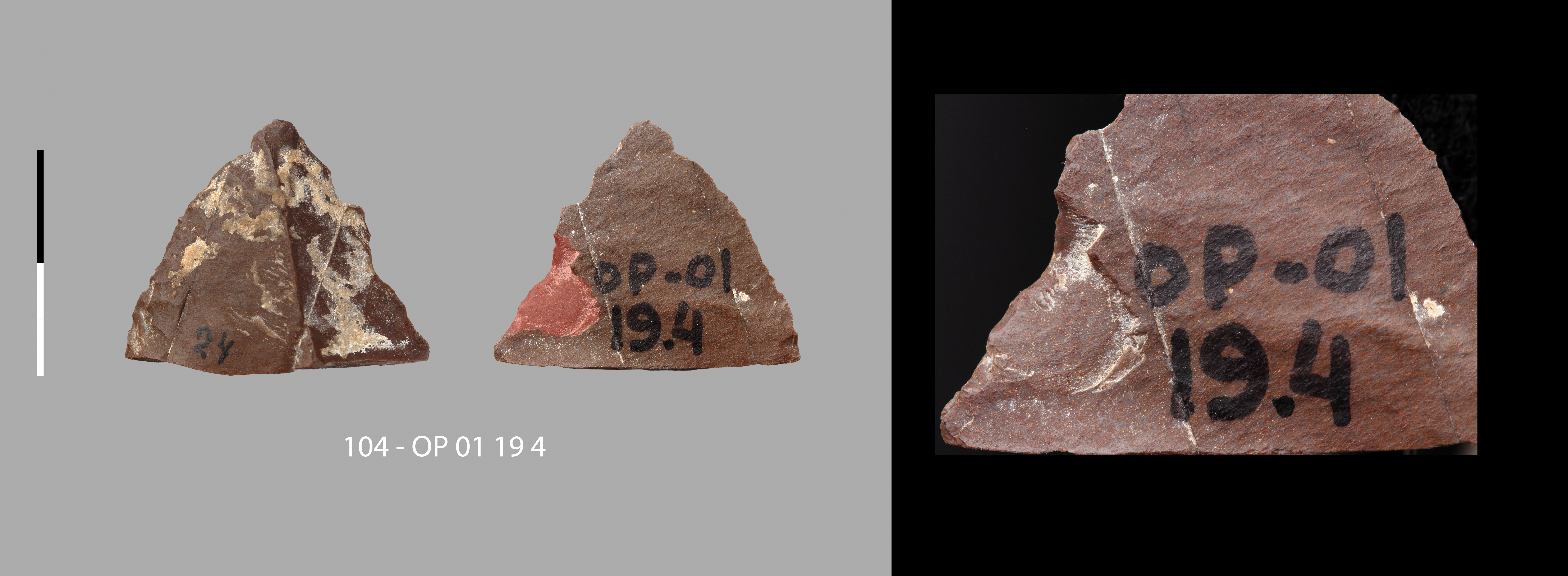

Supplement: S4 Fig — (JPG) [file pone.0328390.s012.jpg]

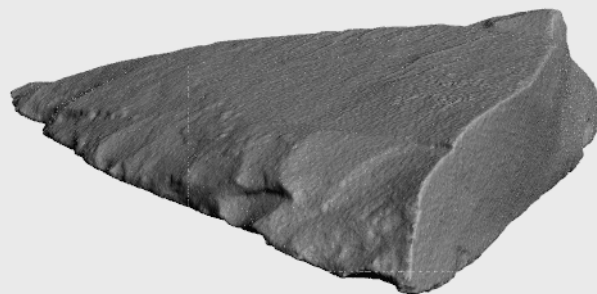

Supplement: S4 File — (PDF) [file pone.0328390.s013.pdf]

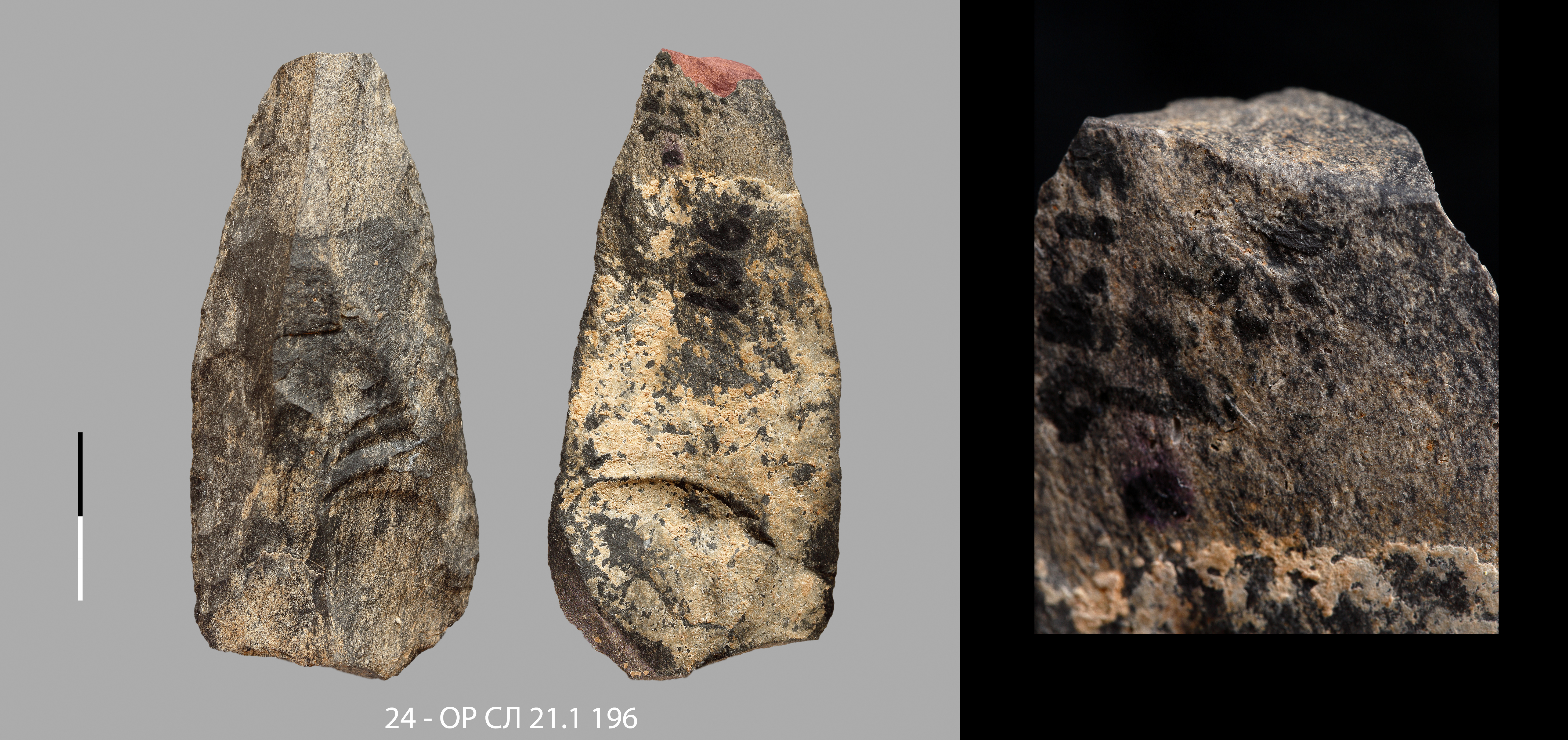

Supplement: S5 Fig — (JPG) [file pone.0328390.s014.jpg]

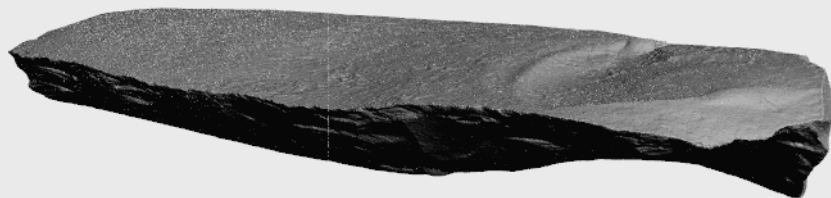

Supplement: S5 File — (PDF) [file pone.0328390.s015.pdf]

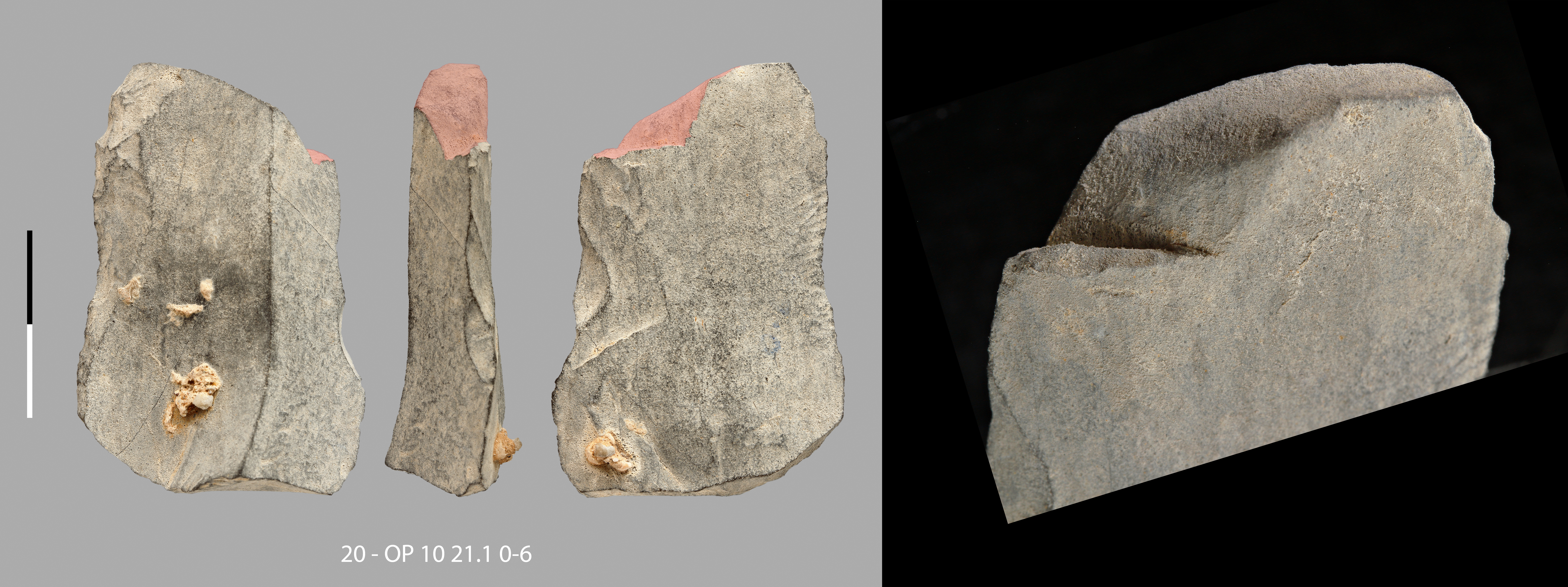

Supplement: S6 Fig — (JPG) [file pone.0328390.s016.jpg]

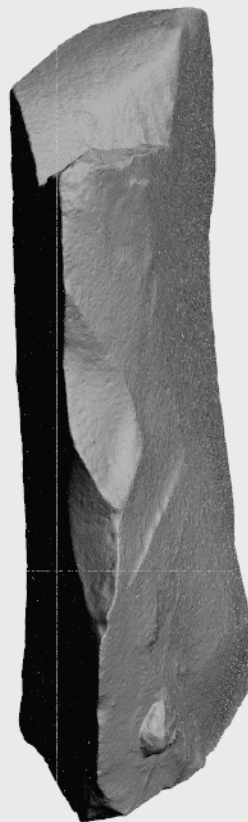

Obi-Rakhmat: 20 - OP 10 21.1 0-6

Supplement: S6 File — (PDF) [file pone.0328390.s017.pdf]

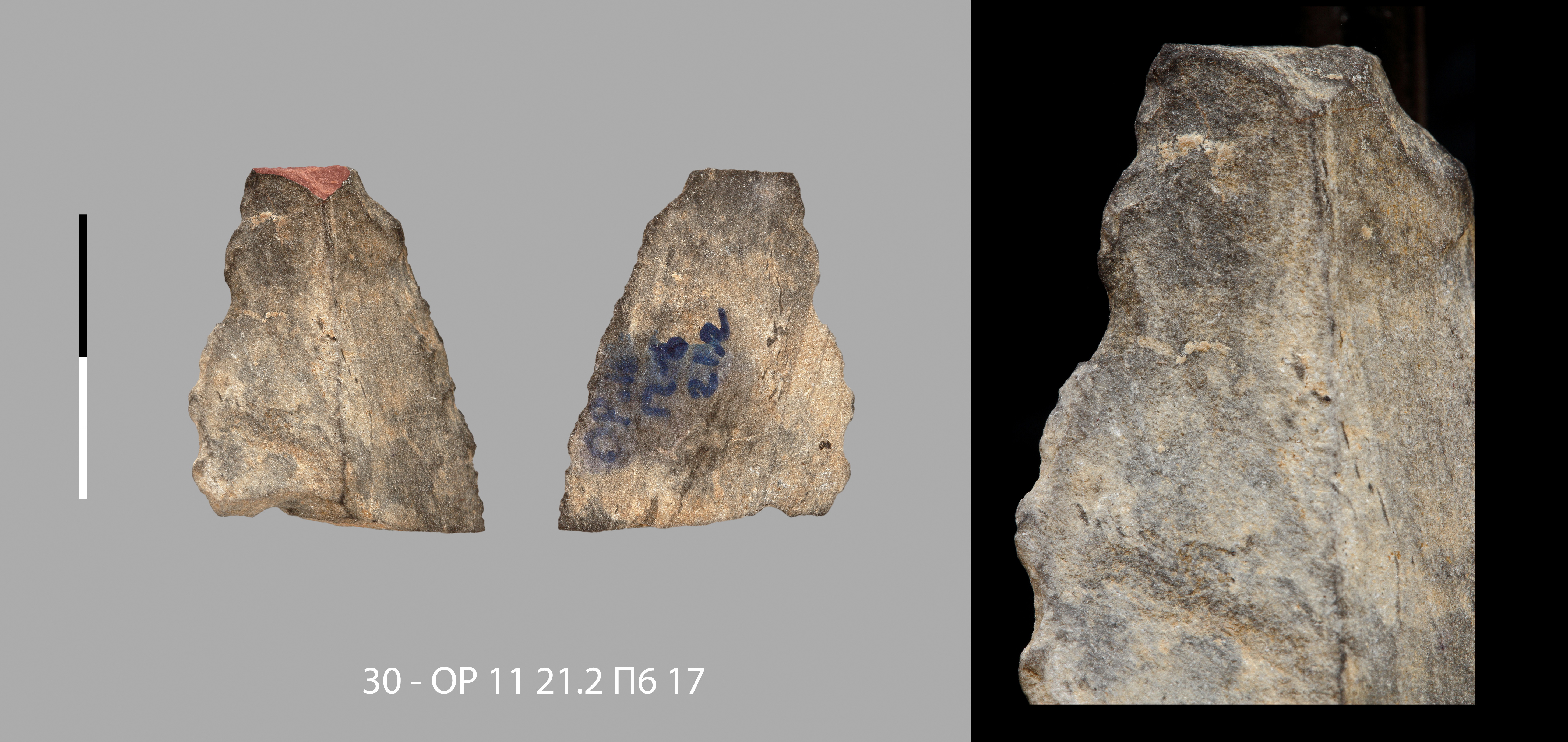

Supplement: S7 Fig — (JPG) [file pone.0328390.s018.jpg]

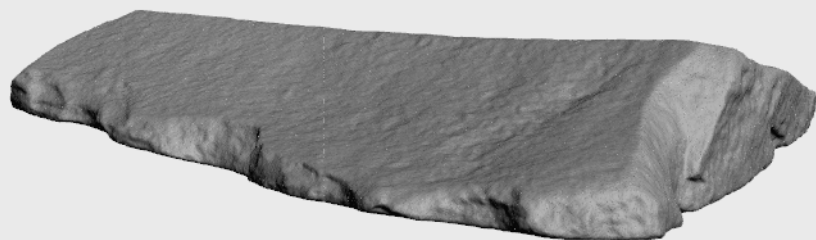

Supplement: S7 File — (PDF) [file pone.0328390.s019.pdf]

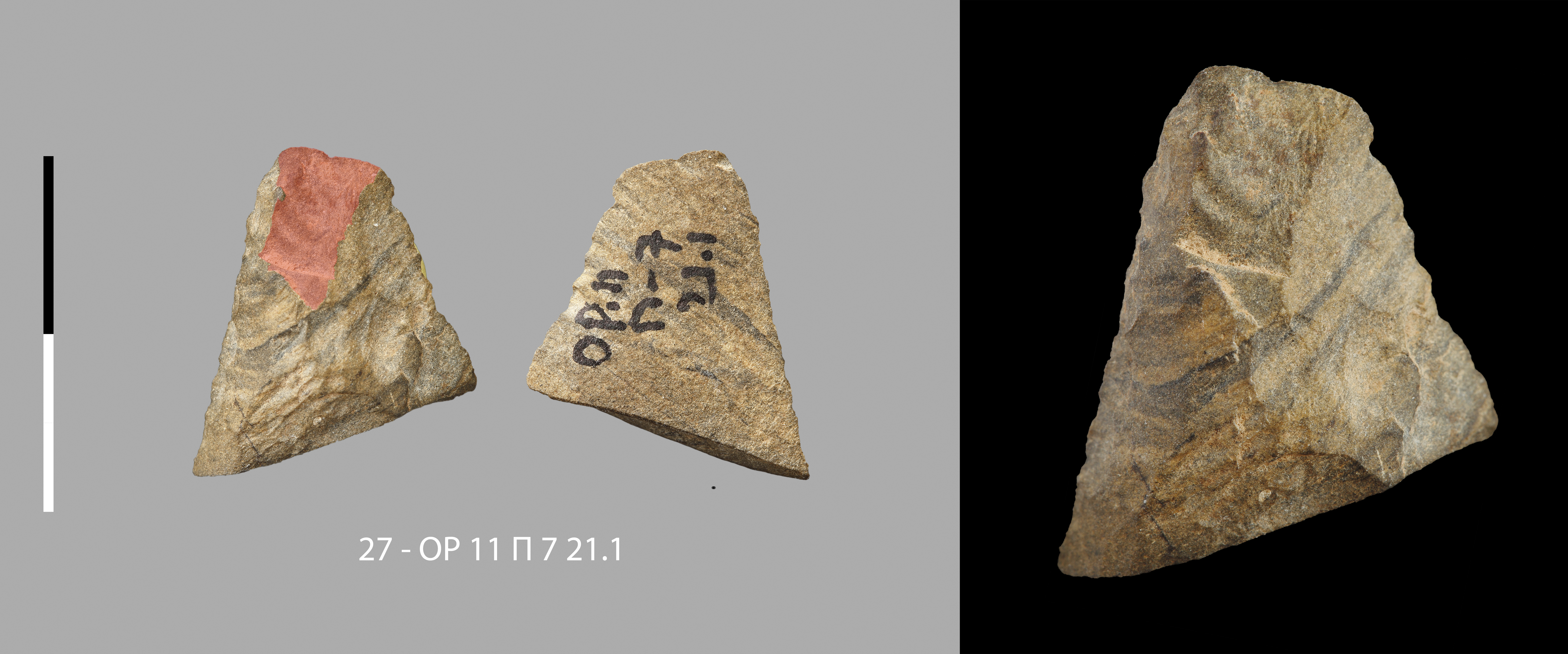

Supplement: S8 Fig — (JPG) [file pone.0328390.s020.jpg]

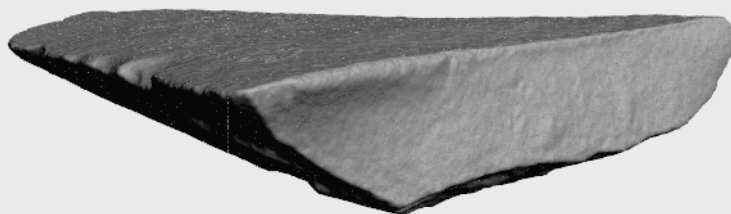

Obi-Rakhmat: 27 - OP 11 П 7 21.1

Supplement: S8 File — (PDF) [file pone.0328390.s021.pdf]

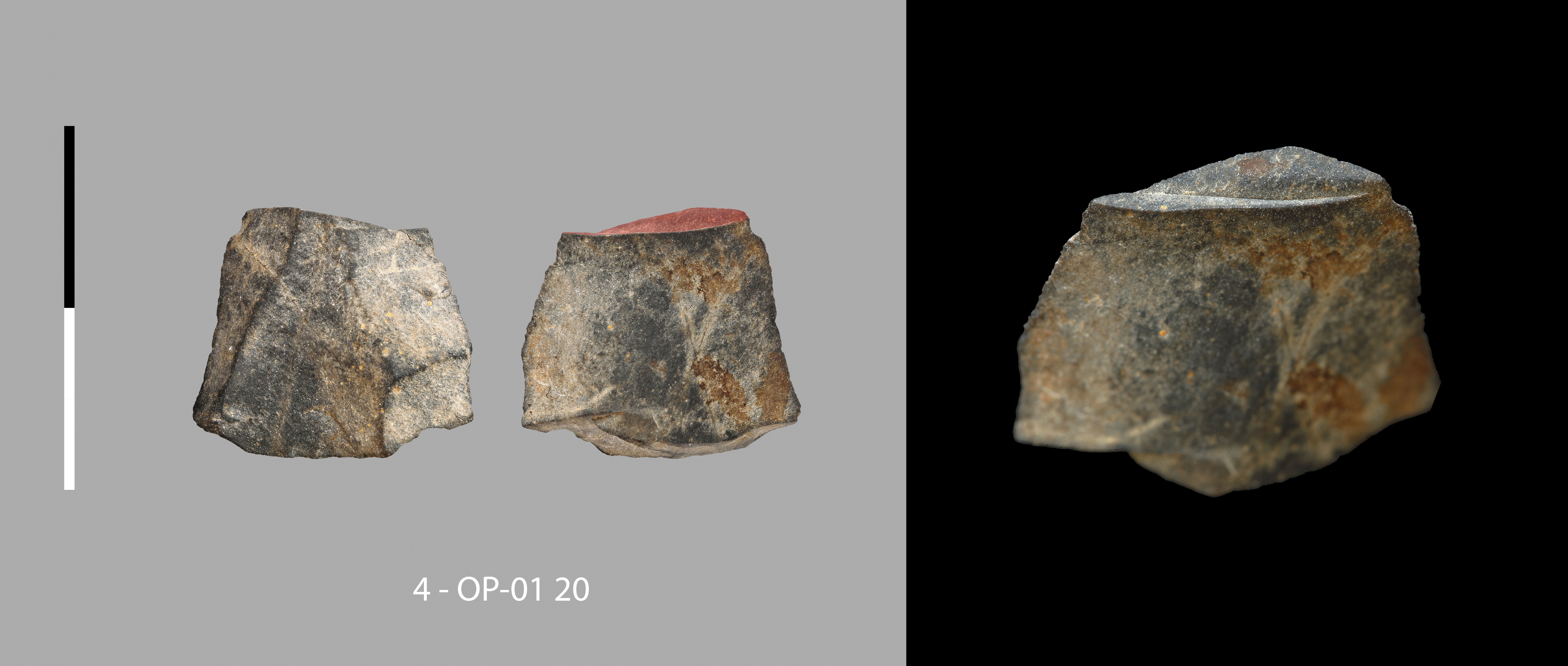

Supplement: S9 Fig — (JPG) [file pone.0328390.s022.jpg]

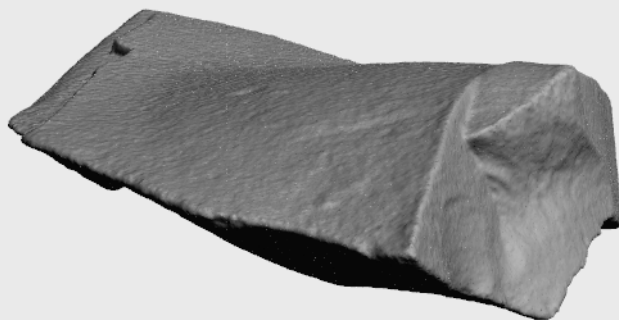

Supplement: S9 File — (PDF) [file pone.0328390.s023.pdf]

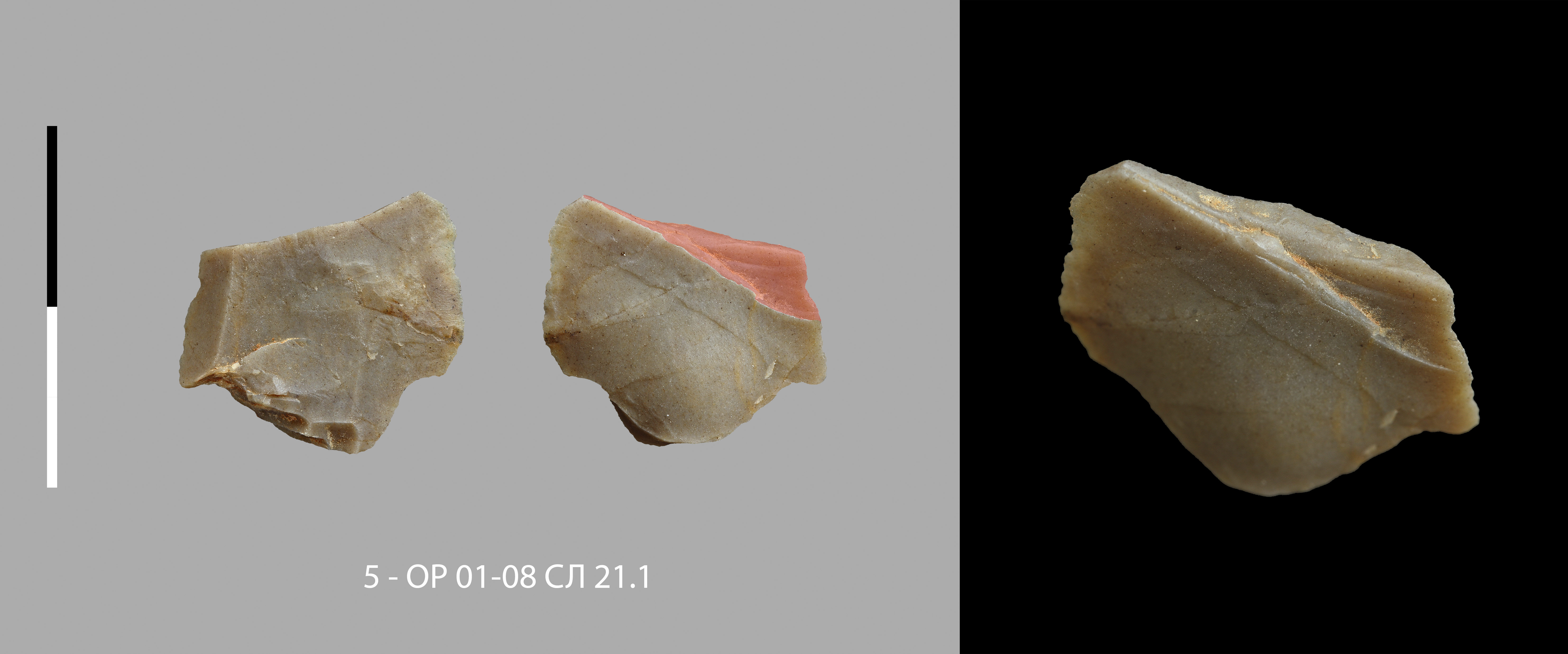

Supplement: S10 Fig — (JPG) [file pone.0328390.s024.jpg]

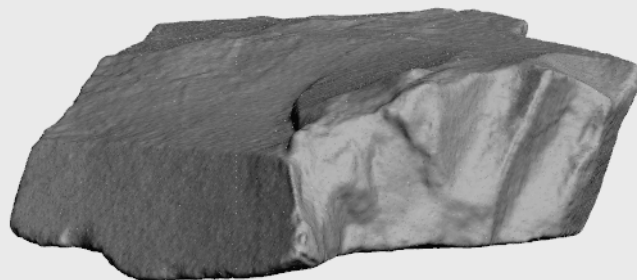

Supplement: S10 File — (PDF) [file pone.0328390.s025.pdf]

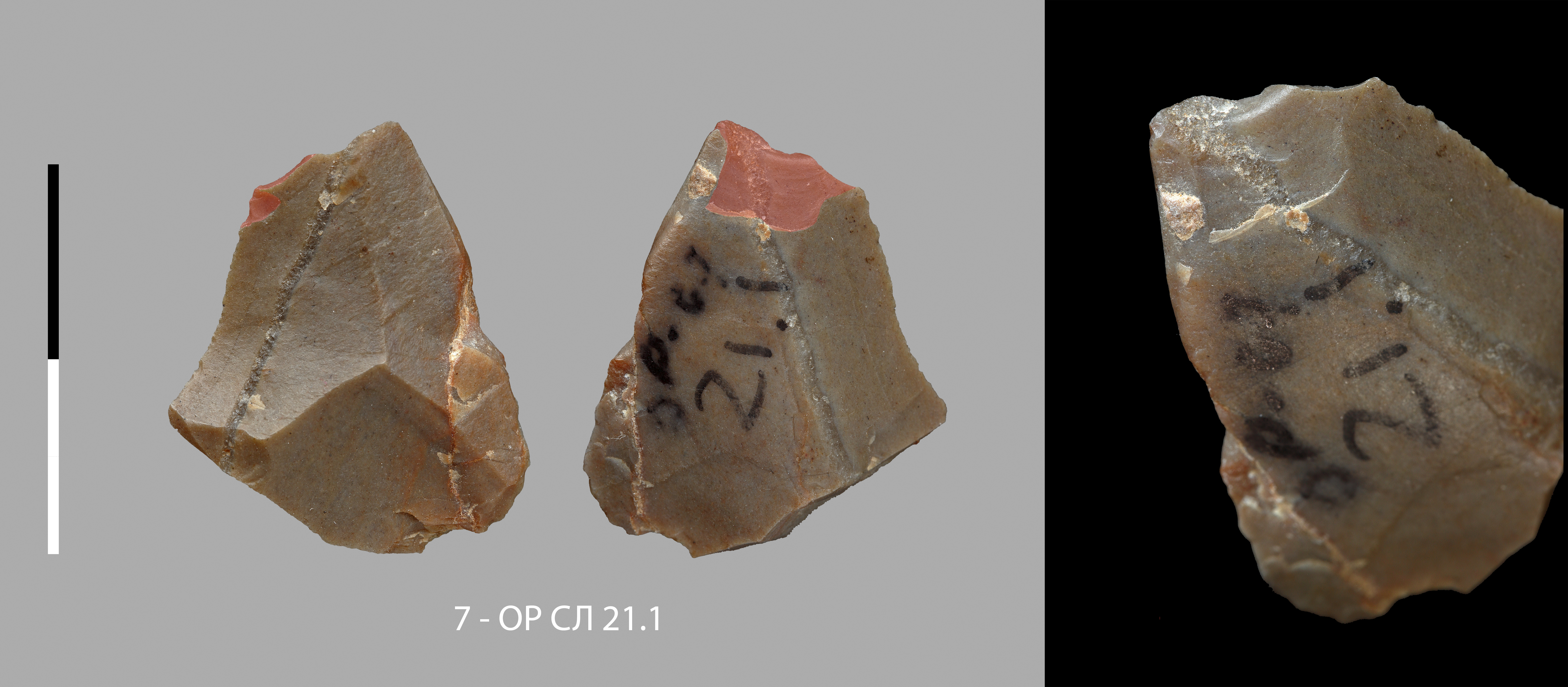

Supplement: S11 Fig — (JPG) [file pone.0328390.s026.jpg]

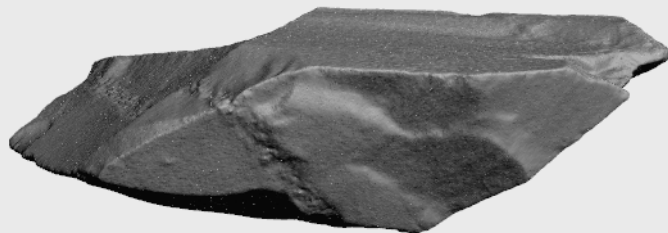

Obi-Rakhmat: 7 - OP CJ 21.1

Supplement: S11 File — (PDF) [file pone.0328390.s027.pdf]

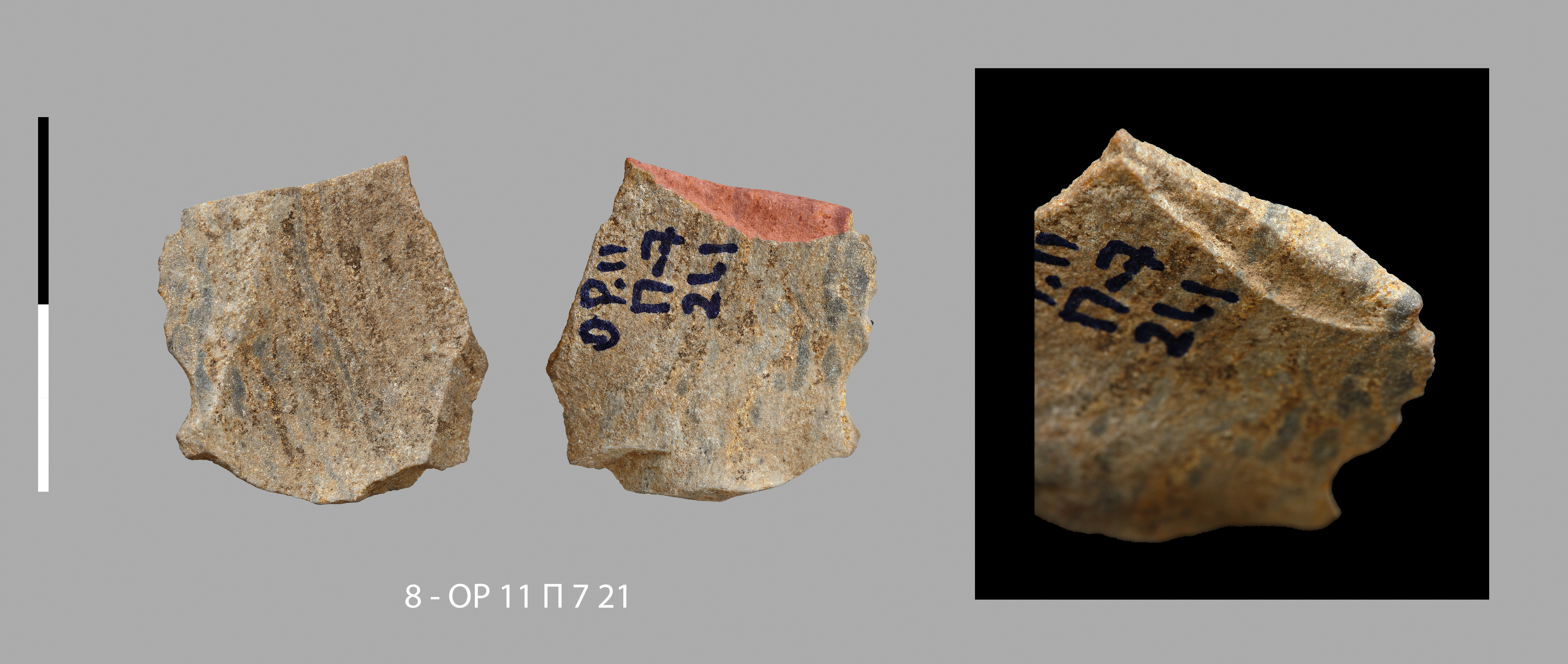

Supplement: S12 Fig — (JPG) [file pone.0328390.s028.jpg]

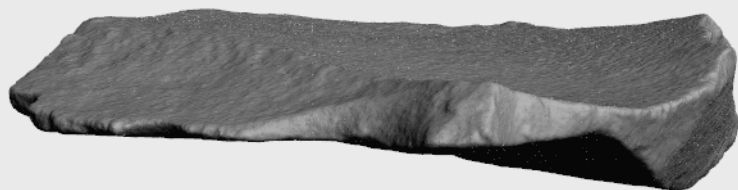

Obi-Rakhmat: 8 - OP 11 П 7 21.1

Supplement: S12 File — (PDF) [file pone.0328390.s029.pdf]

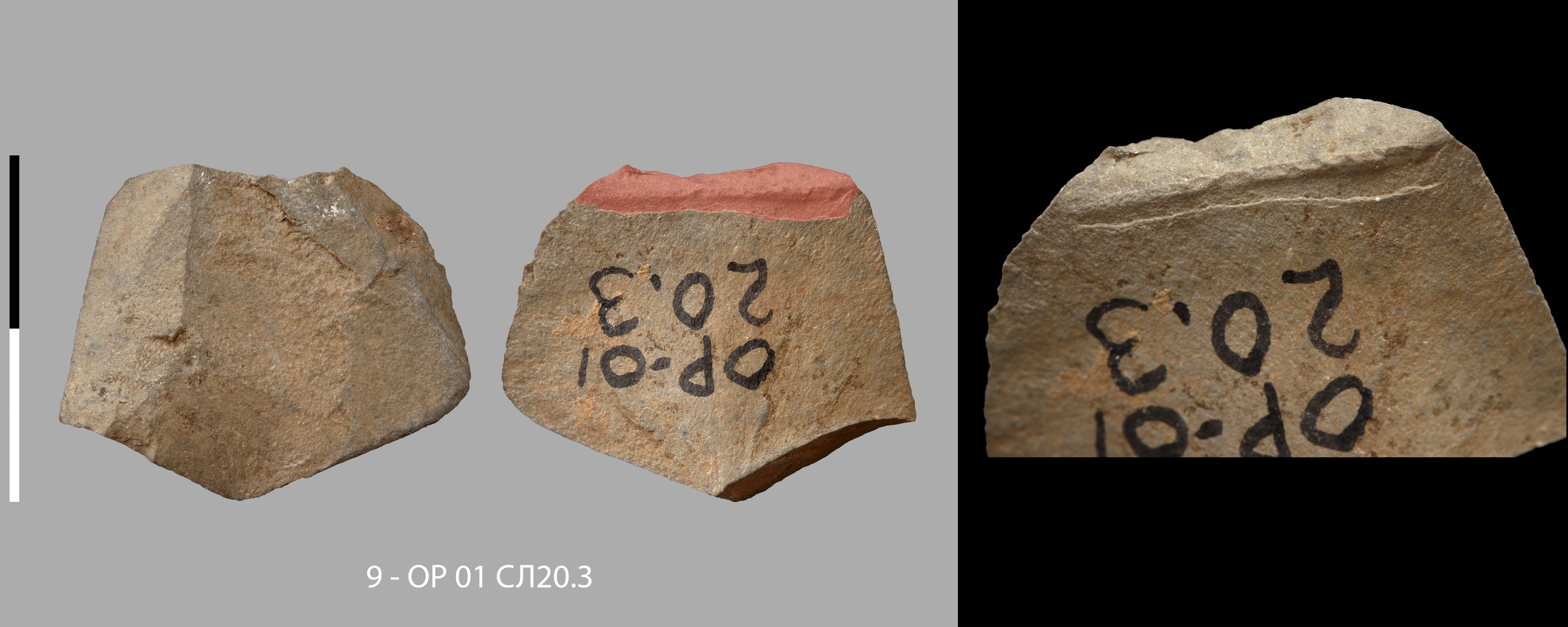

Supplement: S13 Fig — (JPG) [file pone.0328390.s030.jpg]

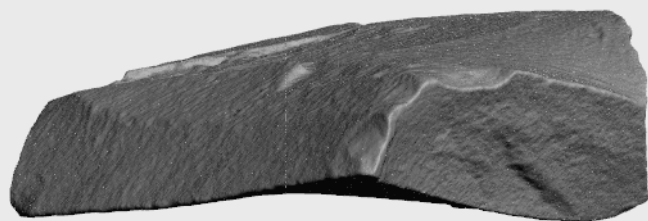

Obi-Rakhmat: 9 - OP 01 С/20.3

Supplement: S13 File — (PDF) [file pone.0328390.s031.pdf]

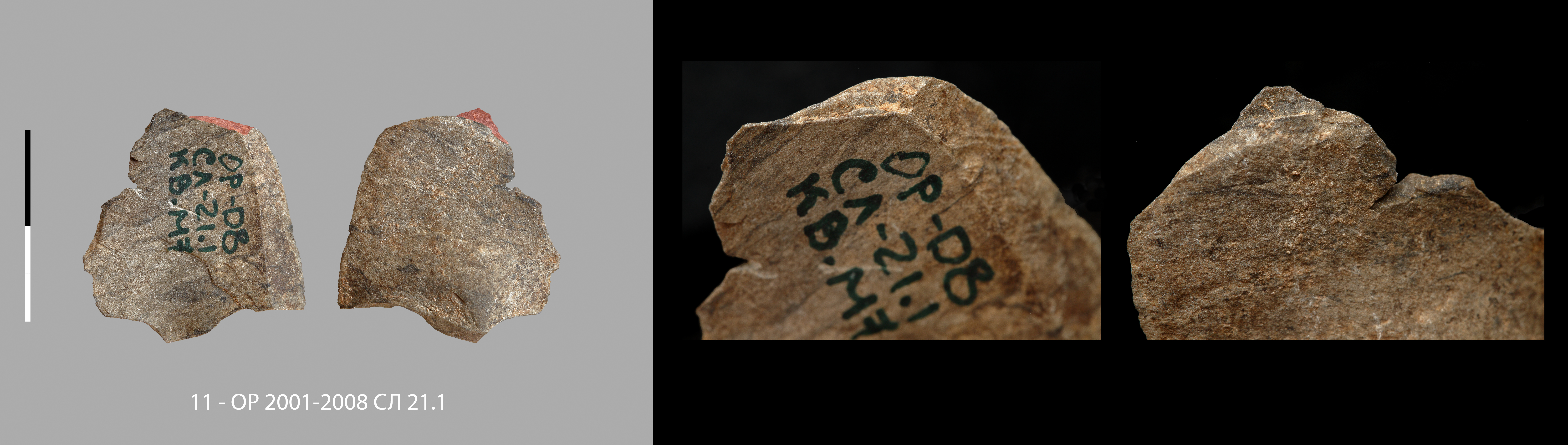

Supplement: S14 Fig — (JPG) [file pone.0328390.s032.jpg]

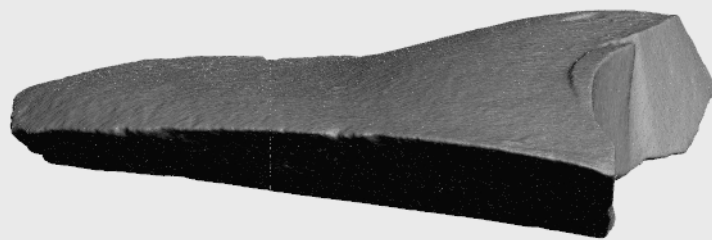

Obi-Rakhmat: 11 - OP 2001-2008 СЛ 21.1

Supplement: S14 File — (PDF) [file pone.0328390.s033.pdf]

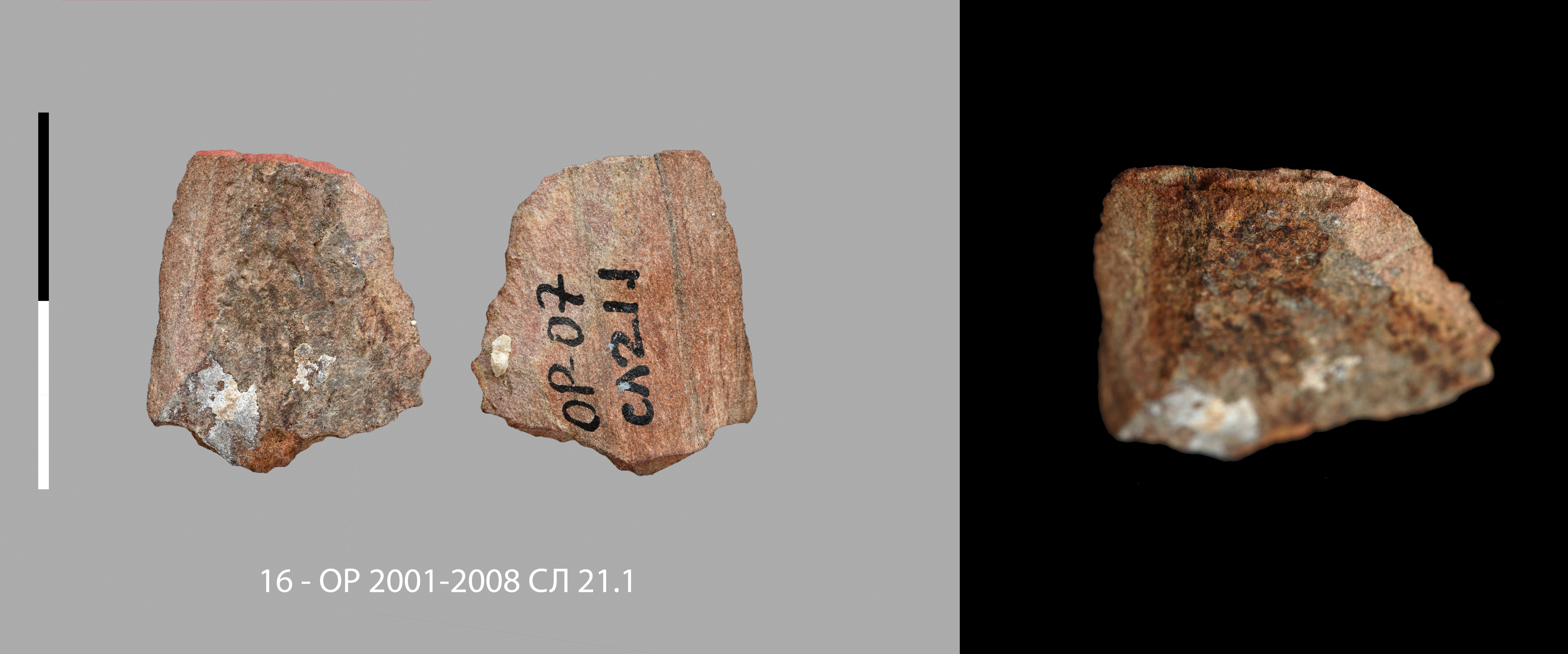

Supplement: S15 Fig — (JPG) [file pone.0328390.s034.jpg]

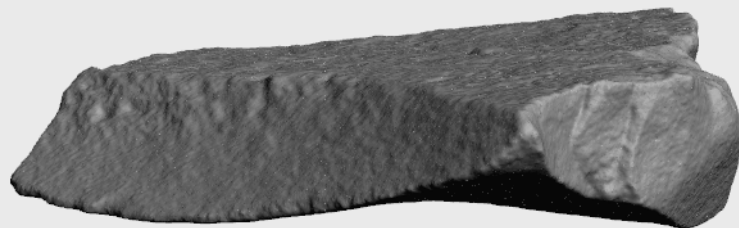

Supplement: S15 File — (PDF) [file pone.0328390.s035.pdf]

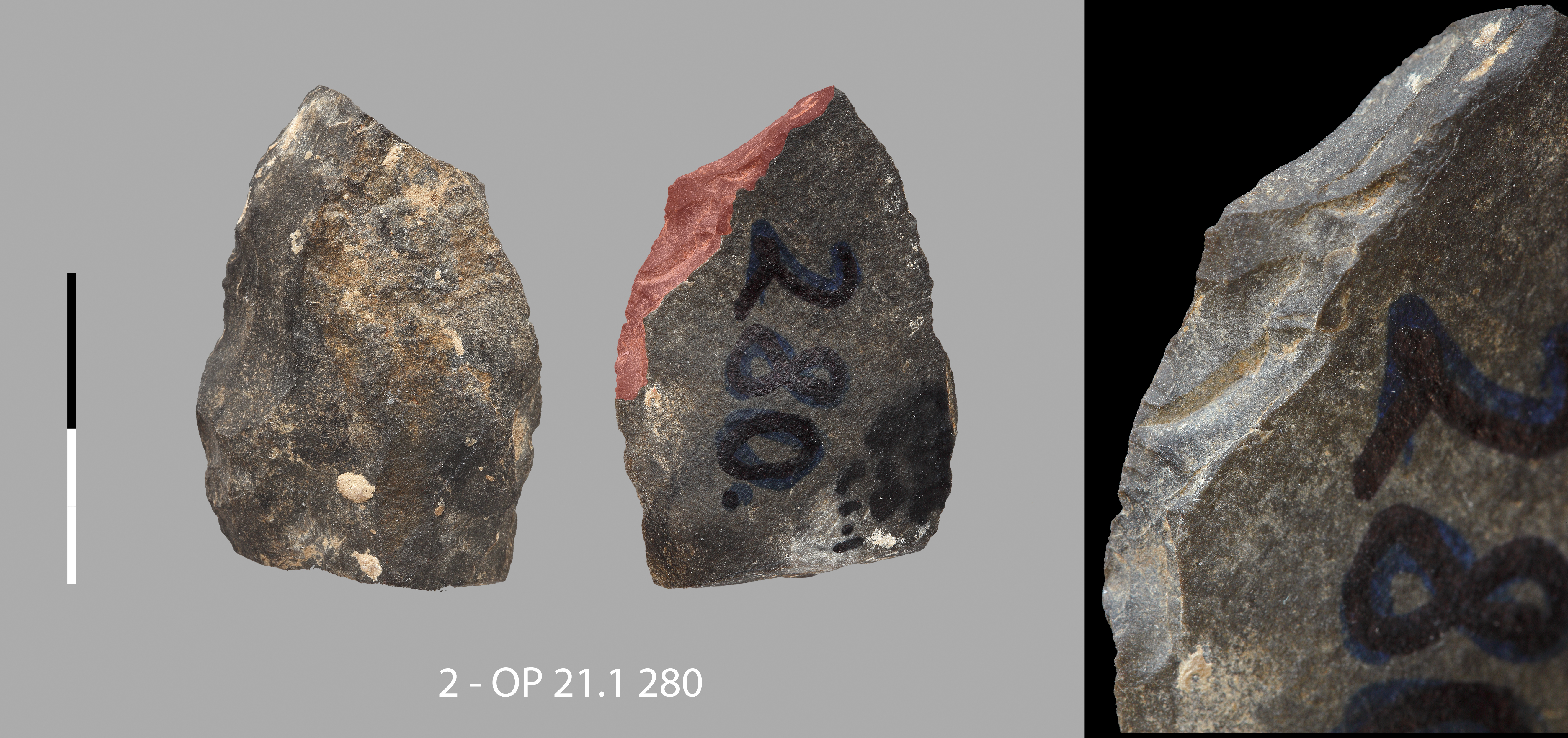

Supplement: S16 Fig — (JPG) [file pone.0328390.s036.jpg]

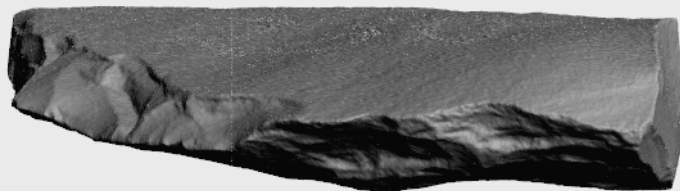

Supplement: S16 File — (PDF) [file pone.0328390.s037.pdf]

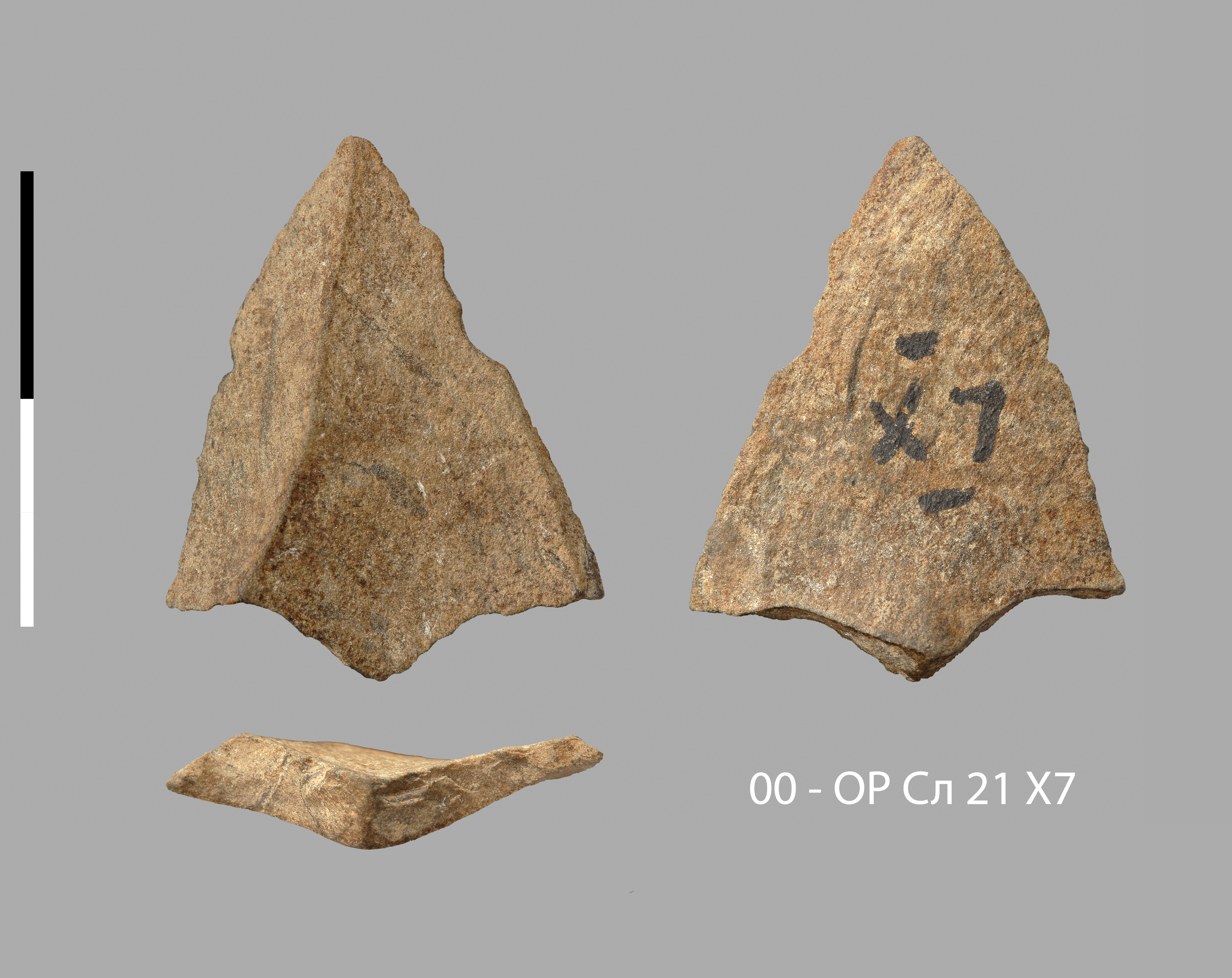

Supplement: S17 Fig — (JPG) [file pone.0328390.s038.jpg]

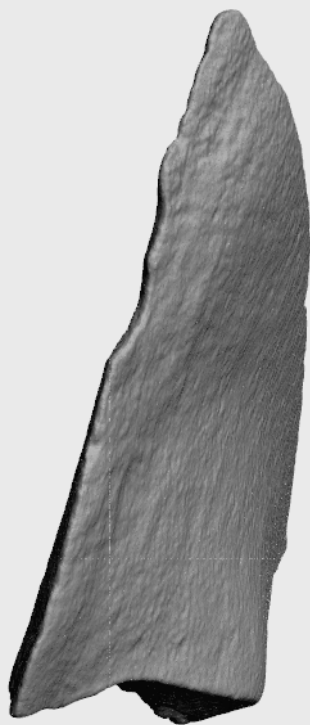

Obi-Rakhmat: 00 - OP-10 21.1 H6 141

Supplement: S17 File — (PDF) [file pone.0328390.s039.pdf]

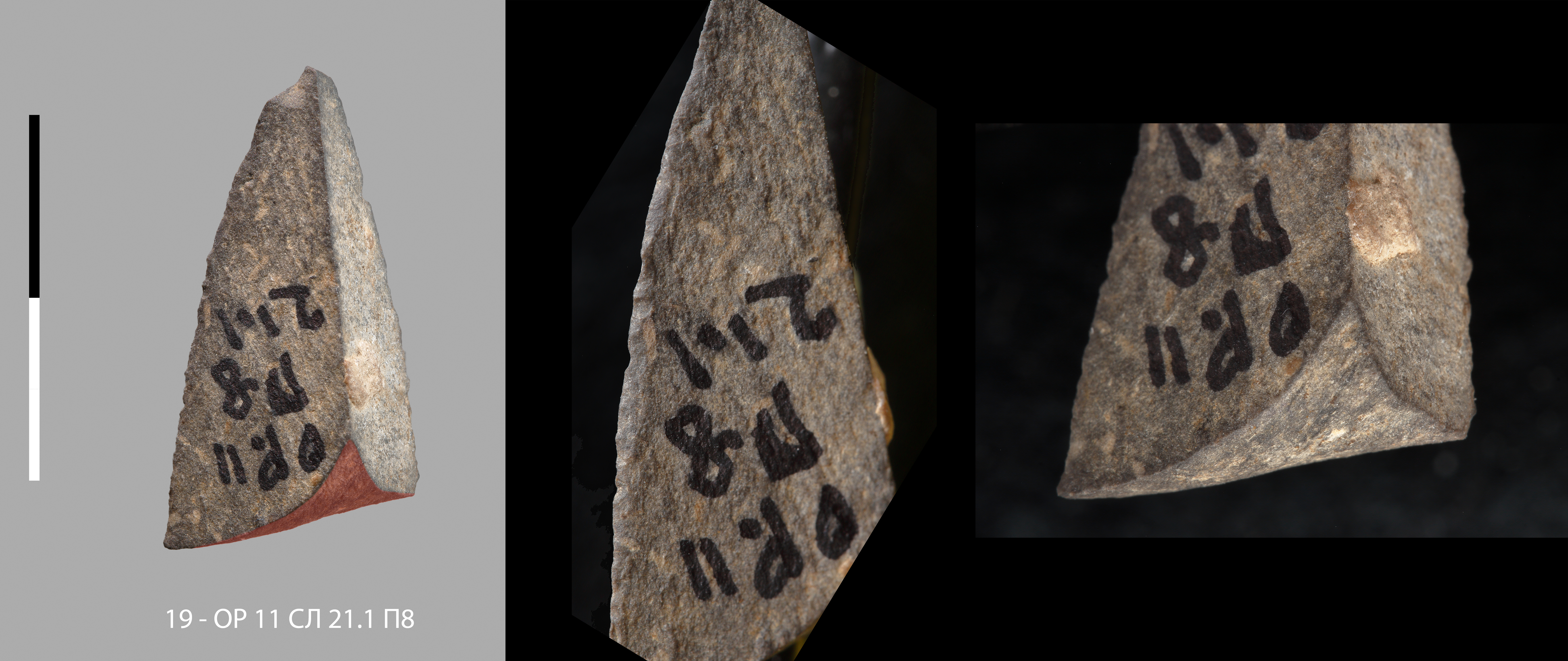

Supplement: S18 Fig — (JPG) [file pone.0328390.s040.jpg]

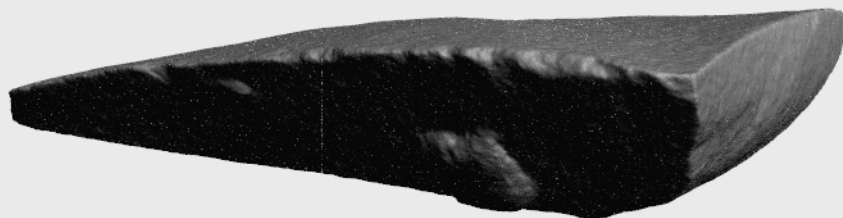

Obi-Rakhmat: 19 - OP 11 СЛ 21.1 П8

Supplement: S18 File — (PDF) [file pone.0328390.s041.pdf]

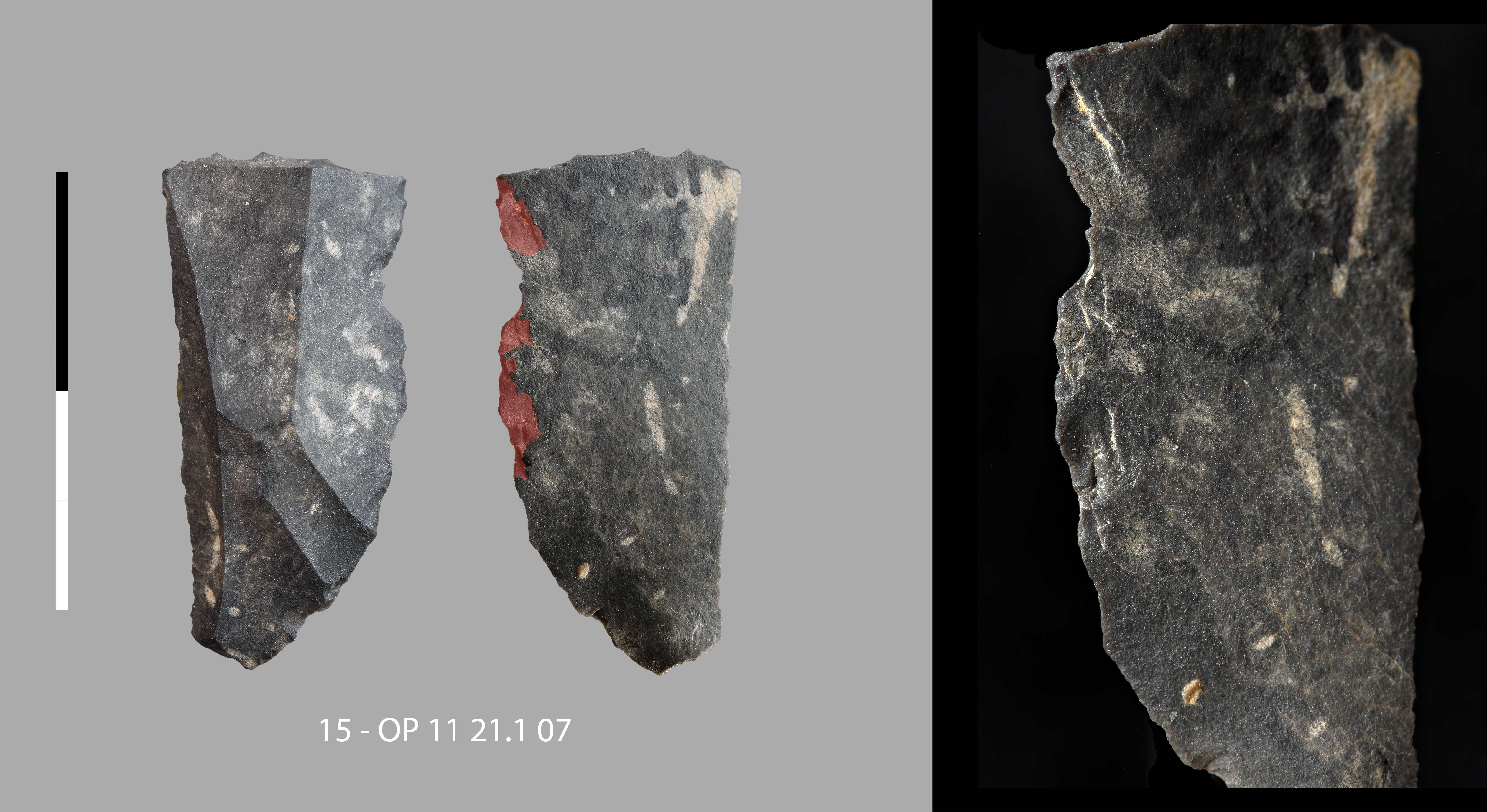

Supplement: S19 Fig — (JPG) [file pone.0328390.s042.jpg]

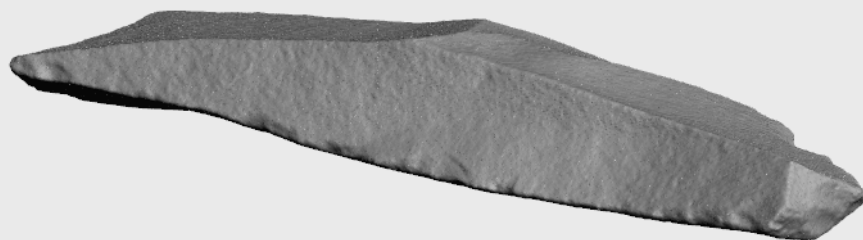

Obi-Rakhmat: 15 - OP 11 21.1 07

Supplement: S19 File — (PDF) [file pone.0328390.s043.pdf]

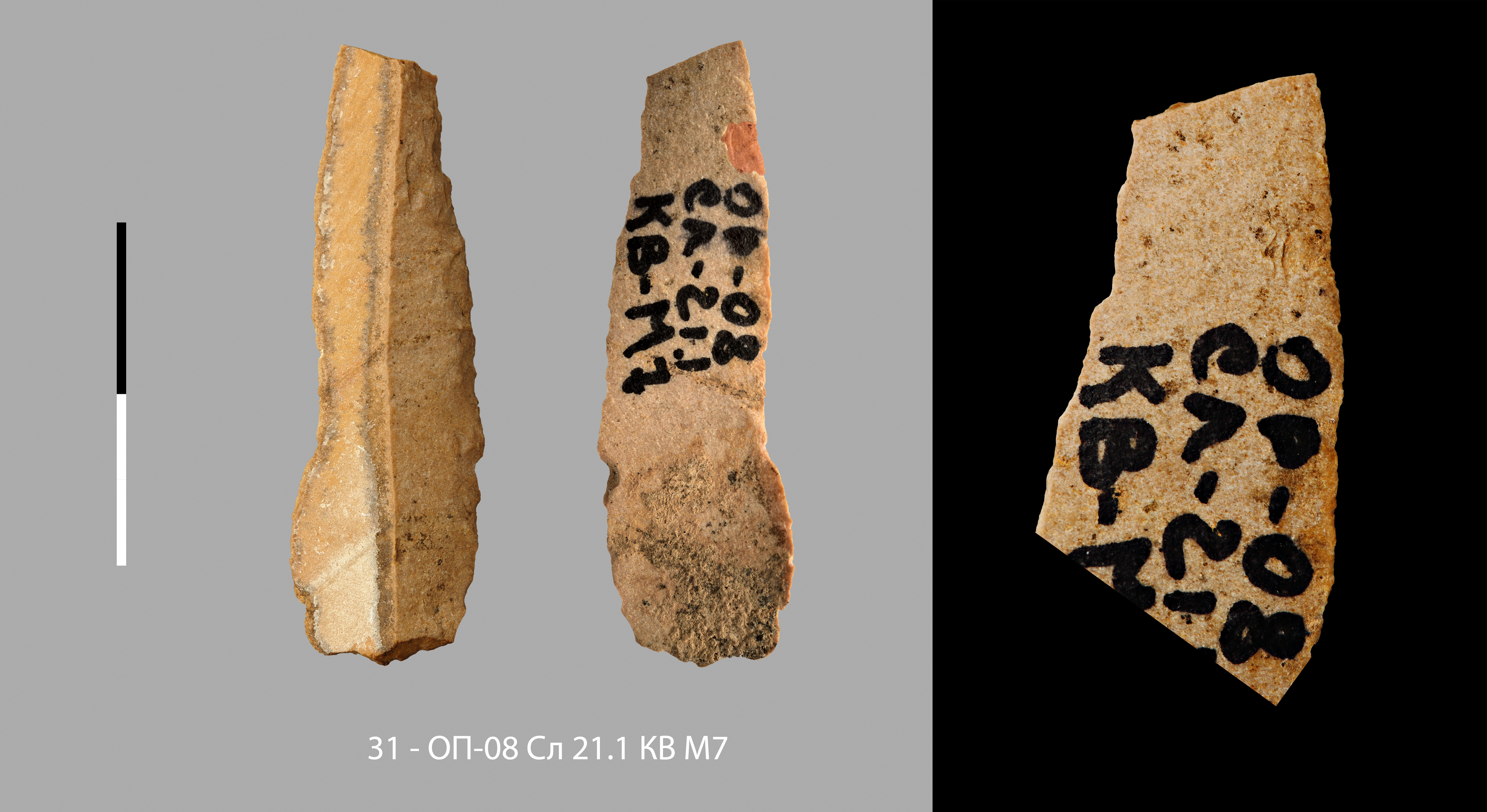

Supplement: S20 Fig — (JPG) [file pone.0328390.s044.jpg]

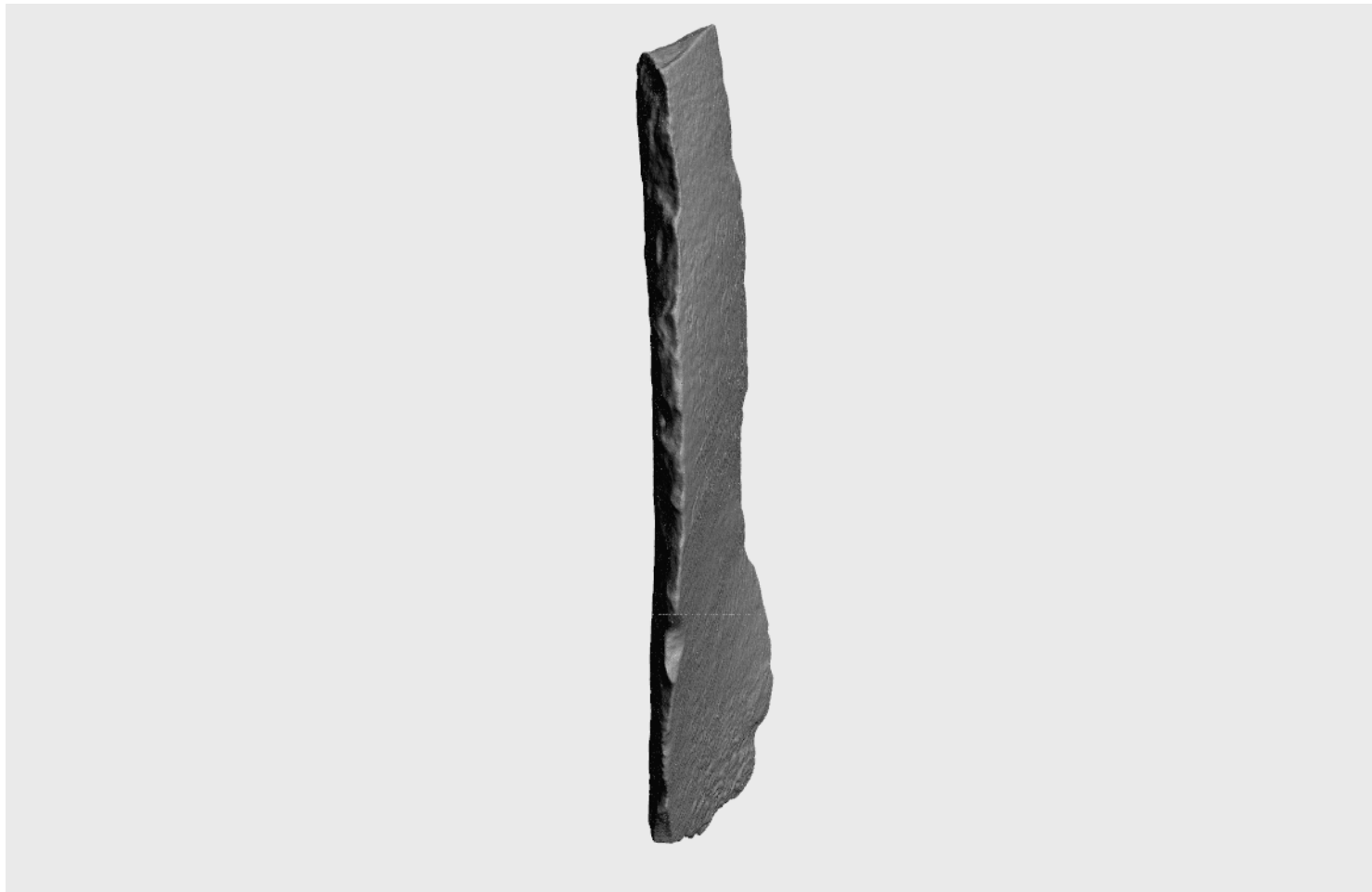

Obi-Rakhmat: 31 - OP-08 Сл 21.1 KB M7

Supplement: S20 File — (PDF) [file pone.0328390.s045.pdf]

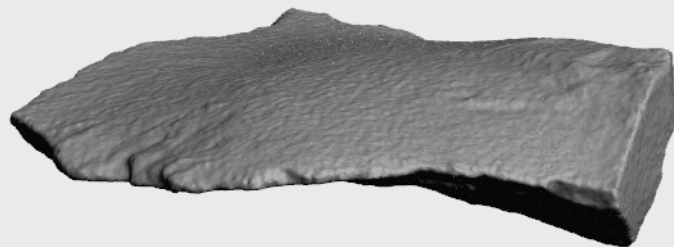

Supplement: S21 File — (PDF) [file pone.0328390.s046.pdf]

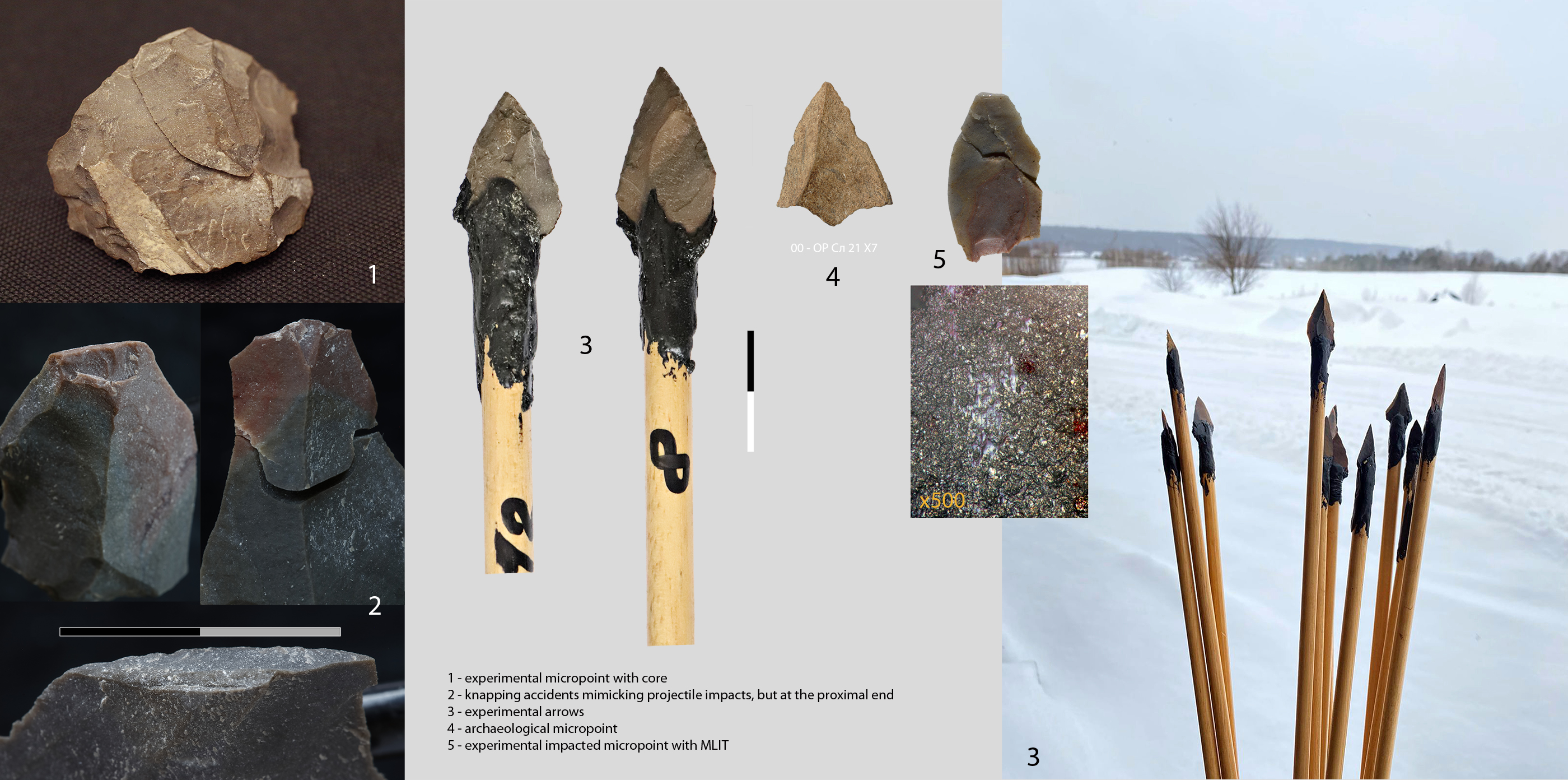

Supplement: S21 Fig — (JPG) [file pone.0328390.s047.jpg]
