# Supplementary material for: Arrow heads at Obi-Rakhmat (Uzbekistan) 80 ka ago?
Source: PLoS One. 2025 Aug 11;20(8):e0328390. doi: 10.1371/journal.pone.0328390 (PMC12338843; doi:10.1371/journal.pone.0328390)
Supplement: Attachment — Submitted filename: PONE-D-25-02378 rev.docx [file pone.0328390.s049.docx]

In the **introduction** the authors declare their goal at line 55 – “We present here the first results of a search for weapon points” and then review the methods that were previously used to do so however by reading through it a notion rises that there is no way of securely doing it. A clear declaration by the authors is needed to show how they are approaching this serious problem:

Lines 70-73: theoretical potentialities with the quote by Bordes – the authors start their review on how arrows can be studies with this claim. I would remove it as this collapses everything that comes later, especially:

Line 97 – “There are two levels of analysis in the identification of a projectile armature” – should add: “to our view” because this is not a conventionality. And: this is given after the example on the Ethiopian points – however the aspects given here can also provide a misleading result as in the case of the Ethiopian points.

And then 119 – “To put it simply, the design of a point will be very different depending on whether you're hunting rabbits with a bow or aurochs with a spear” – do the authors mean here that we do not need these analytical techniques?

153 – goat – better put ibex

**Materials and methods** – in general the chapter needs revision: authors jump from one subject to another and some details are missing. Explanations on the traceological aspect and compared to the technological aspect is not proportional when compared to the body of information given in the results and the discussion clearly shows that the authors do not rely on the results of the trraceological study. It seems that the results are based on the traceological study however this part is applied on a limited number of artifacts and too speculative. On the other hand, the technological study is comprehensive and detailed, providing valuable information on how the points were produced. Other aspects addressed in the discussion such as the comparissong with points from different parts of the world should be mentioned in the methodology

165 – “The analysed sample.... includes typological pieces and a search for triangular shapes” – rephrase...not clear

173 – ~~Photo~~macrographs..and add: micrographs (because a high magnification was used). Was mag of up to 500×? I don’t think that this was used, please specify the mags used in the analysis.

175 – Helicon Focus©

176 - The artefacts were scanned – not clear what is the purpose of this – should be explained in the methodology.

178 – “The macroscopic diagnostic features.... corpus of over 500 flint points and barbs....” on page 134 the authors mention (only one time throughout the publication) that the lithics are of “silicified limestone” – this is a unique material, does it respond to impact the same way? Does MILT form or easy to observe? There is no real discussion on the lithics – fig 7 – looks like flint? Scans look like limestone? Needs further clarification.

182 – “Additional preliminary experiments (S21 Fig)” – are these knapping experiments? The fig is a scan of a point – not clear.

184 – “This step enabled us to observe the knapping accidents” – needs elaboration – how can knapping accident be mistaken by or mimic impact damage.

**Results**

199 – “Traceological inventory” – revise term, first time mentioned in results, not in methodology. This chapter also includes typological asect – should be excluded to a specific sub chapter and better presented.

201 – “we have selected 20 pieces....” – this should appear from the beginning – the article deals with 20 items

202 – “Most of the artefacts” – referring to the 20 selected ones? Not clear

205 – “which depends on the proportion of compressive and bending stress that caused the material to break by buckling or percussion” – what is buckling? Anyway should move to methodology

207 – “the 3D model – these scans do not really allow experts to evaluate the items: scans cannot allow following the orientation of scars, some scans give a very limited view of the artifact (for example – ventral face only). I recommend making a regular plate that illustrate all the 20 points – it will also provide a first wonderful documentation of the artifacts.

209 – fig. 3 – right side annotations: “not relevant, equivocal...” not clear, not explained in the text

217 – fig. 4 – should add a, b, c... – left one – why medium size? Is this the massive point? And are these Abu Zif points?, middle one – Levallois point?, right one – retouched bladelet? – types are not adequately defined.

226 – medium or massive? Inconsistent terminology for the same object?

Fig 6 – impact damage is not clear by looking at these scans, anyway their location should be specified or enlarged.

Fig 7 and 8 – a reference to the location of the MILT shown on the right photo should be marked on the tool. What is the orange colour on the pieces? Is this flint in fog 7?

252 – “It is difficult to distinguish between those accidentally broken during knapping” why? Not clear? Should be explained in the methodology.

250-257 – not clear

268 - **Technological characterization of points production** – the chapter is quite good. However, authors should make sure that the methodology chapter includes the relevant refs for the terms and technique used. A clear explanation to how the typo-technological study was done.

270 - pointed blades or Convergent spalls – inconsistency in terminology?

288 – chain.. - *chaîne opératoire*?

Lines 318-345 in the **discussion** – should move to methodology. The authors should set the stage from the beginning and in the discussion should deal with the results and their contribution.

329 – “...They converge in concluding that the degree of damage is proportional to the energy involved [80,85,86]” – but what is “degree of damage” – not clear

346 – authors conclude: “...much too small to apply any statistical model on traceological criteria – in any case this type of distinction is not done based on statistics. However, in the next lines authors insist on speculating and consistently give the reservations – energy involved is too wide, the poor state of preservation

352 – “We can only note that the points which have the most cutting edges could have been mounted as daggers” – what is “cutting edge” criteria? Is this “longest cutting edge? The author insist on using this criteria however they don’t give the data of this parameter.

336 – “in terms of all the parameters considered” until now it is still not clear which parameters are taken into account (in general according to the refs given and especially in this MS).

Fig 15 – some artifacts appear again

A comprehensive comparison to other sites is given, which is highly important to give the wide chrono-cultural world-wide perspective. This should be mentioned in the methodology too.

**Conclusions –** after the long discussion the authors return to the traceology aspect, which is marginal in this MS.
